# Structural Flexibility and Disassembly Kinetics of Single Ferritin Molecules Using Optical Nanotweezers

**DOI:** 10.1021/acsnano.4c01221

**Published:** 2024-06-08

**Authors:** Arman Yousefi, Ze Zheng, Saaman Zargarbashi, Mahya Assadipapari, Graham J. Hickman, Christopher D. J. Parmenter, Carlos J. Bueno-Alejo, Gabriel Sanderson, Dominic Craske, Lei Xu, Carole C. Perry, Mohsen Rahmani, Cuifeng Ying

**Affiliations:** † Advanced Optics and Photonics Laboratory, Department of Engineering, School of Science and Technology, Nottingham Trent University, Nottingham NG118NS, United Kingdom; ‡ School of Science and Technology, Nottingham Trent University, Nottingham NG11 8NS, United Kingdom; § Nanoscale and Microscale Research Centre, University of Nottingham, Nottingham NG7 2RD, United Kingdom; ∥ School of Chemistry, University of Leicester, University Road, Leicester LE1 7RH, United Kingdom; ⊥ Interdisciplinary Biomedical Research Centre, School of Science and Technology, Nottingham Trent University, Nottingham NG11 8NS, United Kingdom

**Keywords:** ferritin, single molecule, optical nanotweezers, disassembly kinetics, ferritin dynamics

## Abstract

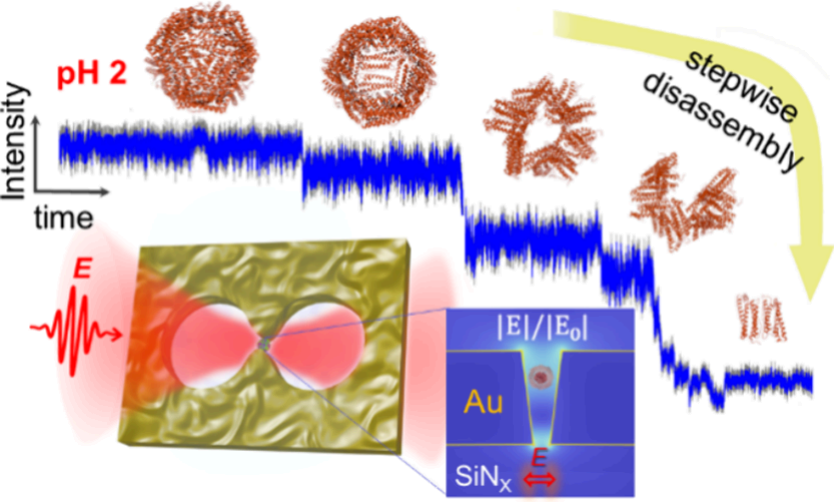

Ferritin, a spherical protein shell assembled from 24 subunits, functions as an efficient iron storage and release system through its channels. Understanding how various chemicals affect the structural behavior of ferritin is crucial for unravelling the origins of iron-related diseases in living organisms including humans. In particular, the influence of chemicals on ferritin’s dynamics and iron release is barely explored at the single-protein level. Here, by employing optical nanotweezers using double-nanohole (DNH) structures, we examined the effect of ascorbic acid (reducing reagent) and pH on individual ferritin’s conformational dynamics. The dynamics of ferritin increased as the concentration of ascorbic acid approached saturation. At pH 2.0, ferritin exhibited significant structural fluctuations and eventually underwent a stepwise disassembly into fragments. This work demonstrated the disassembly pathway and kinetics of a single ferritin molecule in solution. We identified four critical fragments during its disassembly pathway, which are 22-mer, 12-mer, tetramer, and dimer subunits. Moreover, we present single-molecule evidence of the cooperative disassembly of ferritin. Interrogating ferritin’s structural change in response to different chemicals holds importance for understanding their roles in iron metabolism, hence facilitating further development of medical treatments for its associated diseases.

Ferritin is a primary iron storage protein, consisting of a spherical cage formed by 24 identical subunits (24-mer) arranged in threefold and fourfold channels on the shell.^1,2^ Ferritin sequesters iron within a ferrihydrite (Fe^3+^) core and releases it as ferrous iron (Fe^2+^) in response to biological demand. Ferrous iron plays a crucial role in preventing oxidative damage to cells through its participation in the Fenton reaction, which involves the decomposition of hydrogen peroxide and its interaction with reactive oxygen species.^3,4^ Various reductants can facilitate iron mobilization from the ferritin shell through threefold channels.^5,6^ For instance, ascorbic acid (AA) facilitates cellular metabolism and serves as a reducing agent for mobilizing iron in ferritin.^7^ Patients with hemochromatosis, a disorder characterized by significant iron overload, have lower ascorbate levels than normal and, therefore, require supplementation of ascorbate during chelation therapy.^8,9^ High ascorbate levels can cause damage to proteins, lipids, and DNA by generating radicals via the Fenton reaction.^10^ Additionally, high ascorbate concentrations can create an acidic environment, potentially impacting ferritin dynamics and iron release,^11^ as ferritin is known to undergo disassembly at extreme acid conditions (i.e., pH ≤ 2). Disassembled ferritin can reassemble at higher pH due to electrostatic interactions.^12^ These features make ferritin promising for biomedical applications like drug delivery^13,14^ and imaging.^15,16^ However, a deep understanding of its behavior in response to different conditions is essential for effective utilization in these areas.^17^

Various analytical approaches, including gel electrophoresis,^18^ circular dichroism,^19^ ultracentrifugation,^20^ and fluorescence microscopy,^21^ have revealed the reversible disassembly and assembly of ferritin in response to pH changes. The pH-dependent structural alterations and assembly kinetics of ferritin have also been demonstrated in detail using small-angle X-ray scattering (SAXS).^12^ These bulk measurements, however, are limited to providing information about an average response for a protein population.^22^ Recently, using high-speed atomic force microscopy (HS-AFM) and molecular dynamic (MD) simulations, Maity et al.^23^ observed real-time disassembly reassembly of single ferritin molecules under acidic pH conditions. They revealed two disassembly stages: pore formation through the threefold channels and subsequent fragmentation into dimers. This study offers valuable insights into subunit–subunit interactions during protein disassembly. However, certain intermediate steps and small-domain movements in the disassembly process are not fully captured due to the high stiffness of the AFM cantilever.^24^ MD simulation, on the other hand, is restricted by its short simulation time (i.e., ∼ nanoseconds), making it difficult to track large-domain movements of biological entities accurately.^25^ Therefore, a comprehensive understanding of the dynamic and kinetic behavior of single, native ferritin molecules, which link to iron binding, releasing, and interaction with other biomolecules, remains challenging using these methods.

Using plasmonic optical tweezers, our recent work demonstrated the ability to monitor the dynamic changes of individual, unmodified ferritin during molecules *in situ* iron loading.^26^ In this study, we investigated the structural flexibility and disassembly kinetics of single ferritin molecules when exposed to AA (a reducing agent) and an acidic environment. We show that ferritin exhibits increased structural fluctuations at AA concentrations approaching saturation that can be attributed to the large amount of iron release. Additionally, upon exposure of ferritin to acidic conditions at pH 2, the protein became unstable and underwent stepwise disassembly. Using our approach, we tracked the disassembly pathway of single ferritin molecules and the time duration of each intermediate fragment during the disassembly. Furthermore, using a recent application of interferometric scattering called mass photometry (MP),^27^ we confirmed the existence of these intermediate fragments in ferritin at pH 2. Understanding the disassembly process of ferritin could contribute to the crucial initial step in utilizing ferritin for nanotechnology-based applications such as drug delivery and bioimaging.^13,14,17^

## Results and Discussion

### AA Opens Pore Channels of Ferritin

**Figure 1:**
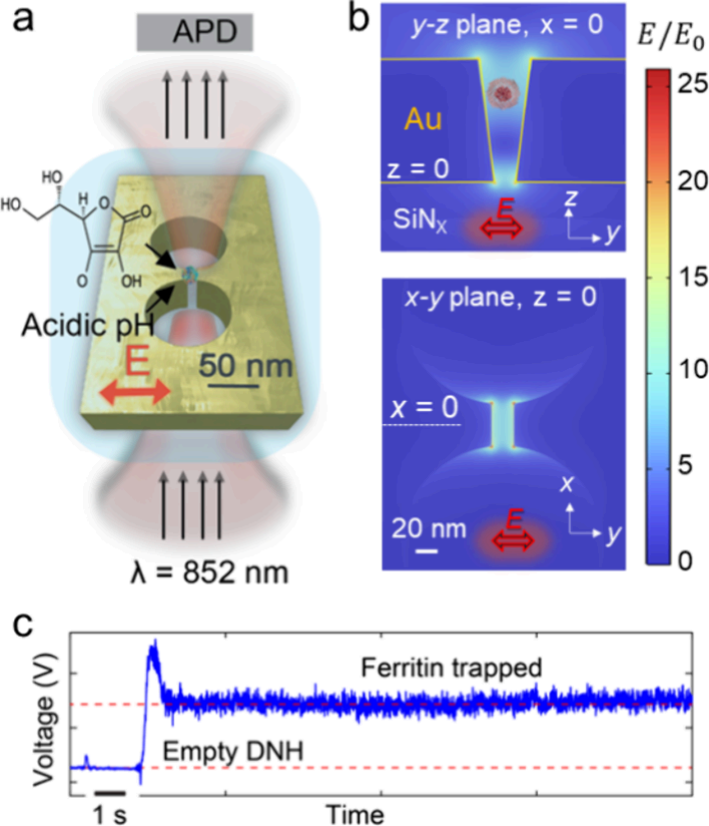
Concept of double-nanohole (DNH)-based optical nanotweezer with the simulated optical field distribution. (a) Schematic representation of a gold DNH positioned within the flow cell (light blue) with an 852 nm laser beam focused on the structure. Solutions of varying pHs and different concentrations of AA are introduced to the trapping site. The transmitted light through the DNH is recorded by an avalanche photodiode (APD). (b) Simulated distribution of electric field enhancement in the DNH structure in the *y*–*z* plane and *x*–*y* plane. (c) Representative transmission trace demonstrating the trapping of a single ferritin molecule.

Figure 1a depicts the double-nanohole (DNH) structure in an aqueous environment within an optical nanotweezer setup, where an 852 nm laser is focused on the structure (see Figure S1 for SEM images of the DNH structures used). The localized surface plasmon resonance generated by the gold DNH highly enhanced the optical field, as shown by the simulated electric field distribution in Figure 1b, with detailed simulation parameters in SI–S2. Subsequently, the tightly confined optical field generates a strong gradient force that enables trapping a nanoparticle inside the gap of the DNH.^28−32^ The presence of the protein in the gap introduces a resonance shift, leading to a change in the transmission detected by the avalanche photodiode (APD). The voltage recorded by the APD responds linearly to the intensity of the transmission. Figure 1c demonstrates a typical trapping trace, where the transmission increases upon trapping a single ferritin molecule due to the dielectric loading.^33−36^

AA reduces ferric iron into ferrous iron, a process that results in an increased production of reactive oxygen species, specifically hydroxyl radicals.^37^ Subsequently, this oxidation process gives rise to the formation of ascorbyl radicals and ferrous ions (eq 1). The ascorbyl radical later reacts with dioxygen, leading to the regeneration of ascorbate and the generation of superoxide ions (eq 2). Both the superoxide and ascorbate radicals interact with ferritin, facilitating the conversion of ferric iron into ferrous iron and thereby promoting the mobilization of iron (eqs 3 and 4)^38,39^:
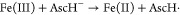
1
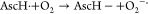
2
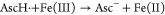
3
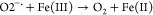
4

**Figure 2:**
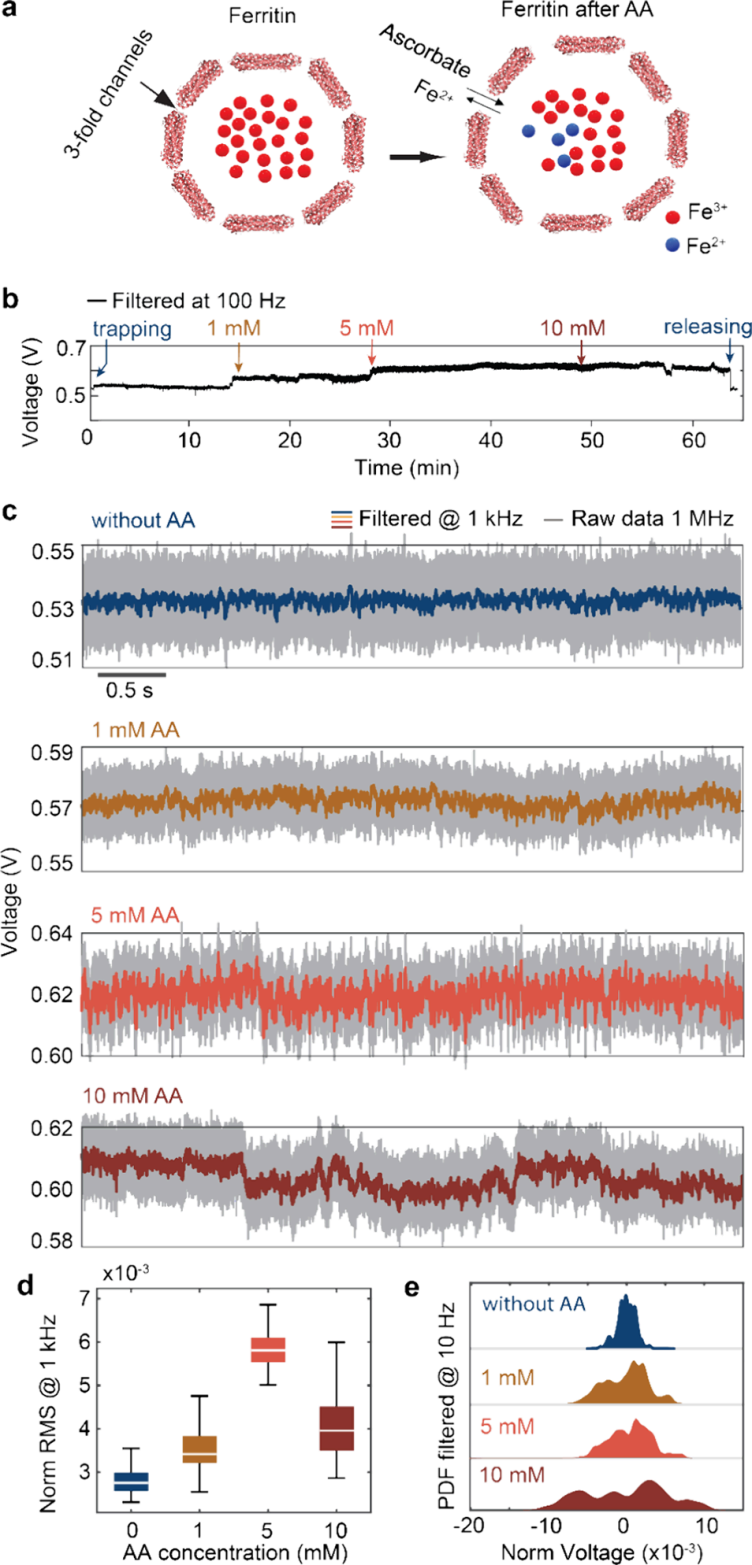
Effect of the AA on the dynamics and iron releasing of ferritin. (a) The cartoon illustrates ferritin before and after exposure to AA and how ascorbate and Fe^2+^ permeate through threefold channels. (b) Continuous transmission traces with a single ferritin molecule trapped in PB buffer, sequentially introducing different concentrations of AA to the trapping site and eventually releasing the ferritin. (c) Five-second transmission traces for the same ferritin in different concentrations of AA. These traces are taken from the trace shown in panel b with positions indicated by arrows. (d) Box plots display the normalized RMS calculated every 0.5 s from a 5-s trace shown in panel c. Data were Gaussian-filtered at 1 kHz. The median value is represented by white lines for each data set, with the box showing the 25th and 75th percentiles. (e) Probability density function (PDF) of 5-s traces of single ferritin molecule and different AA concentrations (filtered at 10 Hz). Data were acquired at 1 MHz and then Gaussian-filtered at 1 kHz and 10 Hz. Significance tests were conducted on the normalized RMS data in this figure (for all values *p* < 0.0001). DNH structure no. 1 was used for this experiment, with its SEM image shown in Figure S1.

Figure 2a illustrates the process of ascorbate diffusion into the ferritin core through the threefold channels,^40^ binding to the ferric ions (Fe^3+^) at the site of the ferroxidase center, leading to the reduction of the ferric ions and eventual release from the protein. Figure 2b,c displays the transmission signals of trapped ferritin when subjected to varying concentrations of AA. As the transmitted light intensity correlates to the conformation of the trapped protein, the root mean square (RMS) of the optical signals reveals the fluctuation of protein conformations.^26^ The RMS of the traces in Figure 2d, along with the repeat experiment in Figure S3, demonstrates increased ferritin dynamics within high concentrations of AA.^41,42^

Introducing 1 mM of AA solution to the trapped ferritin led to a 21% increase in RMS, while a concentration of 5 mM resulted in a 110% increase, indicating an accelerated rate of iron chelation and subsequent release of Fe^2+^ ions.^38^ The increased dynamics arise from the opening and closing of the threefold channels when ascorbate anions pass through and interact with residues like cysteine in the channel.^41,42^ This channel gating behavior is also observed when apo-ferritin is exposed to AA, as shown in Figure S4. At 10 mM, the RMS value remained higher than that of 1 mM but lower than 5 mM. This is attributed to the occurrence of saturation kinetics, whereby the available interactive sites between ascorbate and iron core become limited to generate surface complexes, which aligns with previous work.^38^ The opening of channels promotes the permeation of ascorbate and ferrous iron and consequently accelerates the dissolution of the ferric core.^41^ Reduction of the core, which acts as a ligand for ferritin, destabilizes the protein due to diminished ligand binding.^26^ Indeed, after exposure of ferritin to ascorbate for more than 40 min, the transmission trace (10 mM of Figure 2c) displays slower fluctuations with a larger amplitude, as evidenced by the wider distribution of the probability density function (PDF) at 10 mM AA (Figure 2e).

At high concentrations of AA (1.5 M) when the solution becomes strongly acidic, we observed that ferritin underwent disassembly (Figure S5a). It takes approximately 12 min for the ferritin to be fully disassembled and escape from the trap in a stepwise fashion. During this time, the RMS value increased due to the unfolding of the ferritin channels (Figure S5b–d).^23,39^ Moreover, the presence of 1.5 M AA resulted in a pH value of around 2, creating an acidic environment that further contributed to the disassembly of ferritin. To identify the impact of an acidic environment on ferritin's structure, we focused on the conformational dynamics of single ferritin molecules subjected to varying pH conditions *in situ*.

### Tracking Disassembly Kinetics and Fragmentation of Single Ferritin Molecules

**Figure 3:**
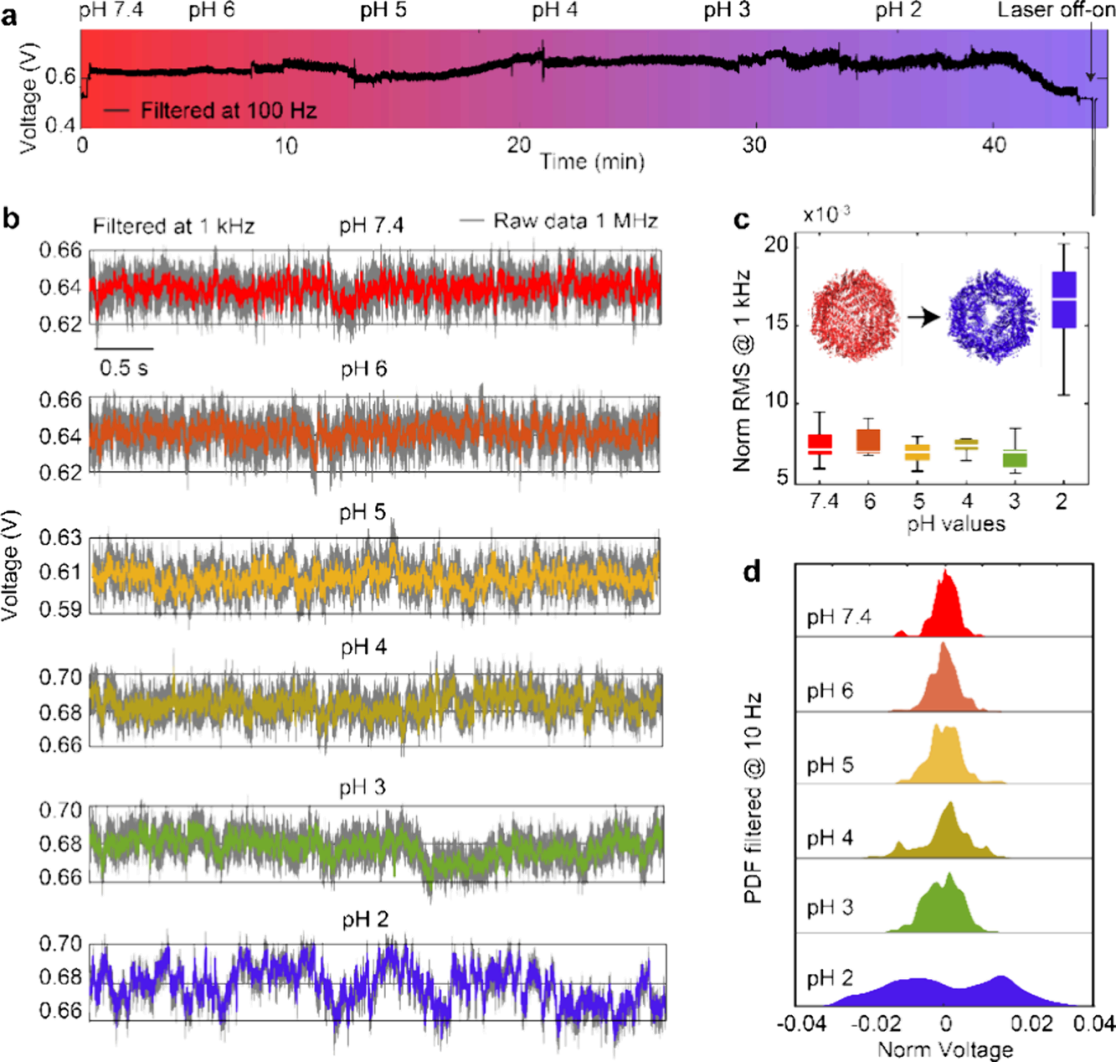
Conformational dynamics of trapped single ferritin molecule exposed to solutions with different pH values. (a) Continuous transmission trace of a DNH with a ferritin protein trapped at pH 7.4 and subsequently introducing solutions of pHs of 6, 5, 4, 3, and 2 to the trapping site. After exposure to pH 2, the transmission signal decreased over time and eventually returned to the baseline. (b) Five-second transmission traces of the same ferritin molecule exposed to solutions with pH values of 7.4, 6.0, 5.0, 4.0, 3.0, and 2.0 in sequence. (c) Box plots display the 25th and 75th percentiles of the normalized RMS values calculated every 0.5 s from 5-s traces in panel b. The inserted cartoon illustrates the opening of the threefold channels upon introducing to pH 2.0 buffer. Significance tests were conducted on the Norm RMS data. The *p* value for comparisons between pH 2 and the other pH values was less than 0.0001 (*p* < 0.0001), while comparisons between other pH values are not significantly different. (d) PDF of 5-s transmission traces (filtered at 10 Hz) for single ferritin molecule exposed to different pH values (panel b). Data were acquired at 1 MHz sampling rate and then Gaussian-filtered at 1 kHz (panels b and c) or 10 Hz (panel d). DNH structure no. 1 was used for this experiment, with its SEM image shown in Figure S1.

To understand the effect of pH on ferritin dynamics, we trapped a single ferritin molecule at pH 7.4 and replaced its environmental solution with an acidic pH (see the methods section) sequentially from 6.0 to 2.0 (see transmission traces in Figure 3a,b). The normalized RMSs of the traces at pH 7.4 to 3.0 are relatively stable with values between 0.005 and 0.01 (Figure 3c), suggesting a stable structure within these solutions. Ferritin comprises 24 subunits categorized as heavy (H) or light (L), with the L-chain being more stable due to hydrogen bonding and salt bridges among subunits.^12,43^ The equine spleen ferritin used in this work has a relatively stable structure at acidic pH due to the dominant L-chain subunits.^2,12,43^ However, upon changing the pH to 2.0, the RMS jumps by 140%, indicating that the protein becomes highly dynamic (Figure 3c). The mechanical instability of ferritin at pH 2.0 is attributed to protein swelling, monomer rotation, and subsequent dimer movement within the threefold channel, leading to channel opening.^12,23,44^ This channel opening may facilitate the dissolution of the ferrihydrite core before disassembly, as reported previously.^23,45,46^

**Figure 4:**
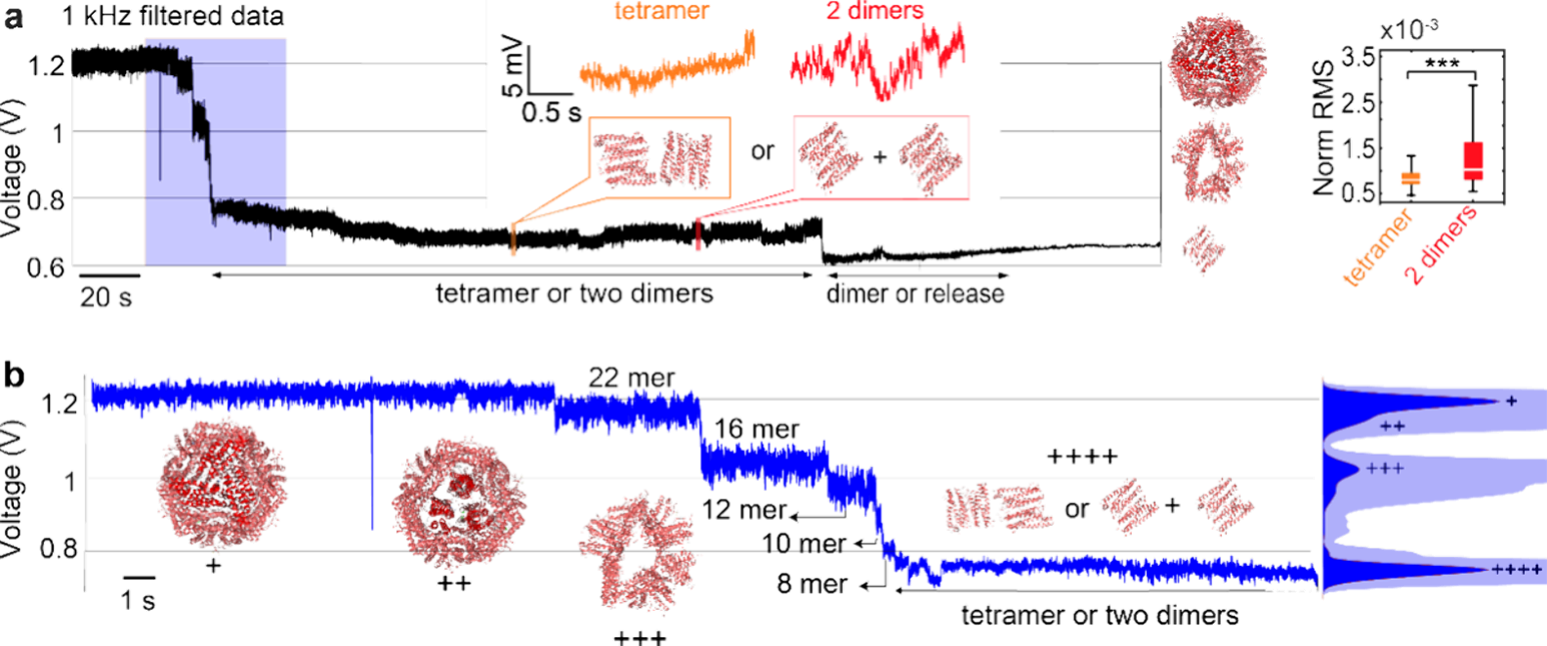
Kinetics of a single ferritin molecule disassembling into its subunits. (a) Transmission trace with multiple drops demonstrating the full disassembly of a single ferritin molecule upon exposure to pH 2.0. Insets provide magnified traces potentially indicating the presence of one tetramer (orange) or copresence of two dimers (red) in the trapping site, along with their normalized RMSs shown on the right (asterisks *p* < 0.005 on Norm RMS). The crystal structures on the right (PDB: 1IER) represent the structures corresponding to the transmission levels (b) A zoomed-in trace from the blue-shaded region of the panel focusing on the fragmentation process of ferritin. The “+” marks the levels corresponding to the fragments of 24-mer, 22-mer, 16-mer, and tetramer or two dimers, matching with the peaks of the PDF on the right (with the transparent blue as the magnified PDF). DNH structure no. 2 was used for this experiment, with its SEM image shown in Figure S1.

When exposed to the acidic solution for a long duration, ferritin subunits began to dissociate, causing protein disassembly. Figure 4a demonstrates the capability of our approach to track the full disassembly kinetics of ferritin after the chamber is filled with pH of 2.0. The stepwise reductions in the transmission signal (Figure 4b) show the step-by-step disassociation of ferritin's subunits. The molecular weight of the fragment remaining in the trap is reduced during this process, which consequently affects the transmission signal. When a DNH traps a single protein, its transmission is influenced by the protein’s refractive index, which is determined by the protein’s polarizability.^47^ Three parameters contribute to the polarization of the single protein: the dielectric constant, volume, and shape.^48^ The ferritin subunits in this work have identical dielectric constants due to their predominantly similar L-chain compositions.^43,48^ Additionally, the volume of oligomers is linearly correlated with their molecular weights since their mass density is uniform. When approximating the protein shape as spherical,^28^ we expect a linear relationship between the particle volume and the transmitted signal of DNH structures, as previously reported.^28^ This linear relationship is further confirmed by the finite element method (FEM) simulation shown in Figure S2. This data show that we can follow the ferritin disassembly process quantitatively.

The maximum transmission (*V*_24mer_) during the disassembly process represents the signal from a fully assembled ferritin with 24 subunits, and the transmission level at which the protein is released was labeled as *V*_DNH_. We linearly normalized the transmission (APD signal) by taking the signal of trapped ferritin (*V*_24mer_) to be 100% and the signal of the empty DNH (*V*_DNH_) to be 0%. By taking a sphere approximation, each subunit contributes to 4.17% of the optical signal. With this assumption, we correlated each drop of the APD signal to the dissociation of a certain number of subunits. In this study, we identified the disassembly steps based on the observation that the transmission signal deviated from and fell below its mean value. A zoomed-in view of these disassembly steps is provided in Figure S6 of the Supporting Information.

Table 1 lists the obtained mean values of APD signals of each level in Figure 4, their normalized value, and the estimated number of subunits. We first observed a drop of 10%, corresponding to the dissociation of a dimer, leaving the ferritin to be a 22-mer subunit. Following this, the disassembly procedure continues by reduction of the trapping signal by 30, 45, 70, 80, and 95–100%, which is attributed to the 16-mer, 12-mer, 8-mer, tetramer, and dimer, respectively.

**Table 1:** Disassembly of a Single Ferritin Molecule upon Exposure to pH 2.0

*V*_mean_ (mV)	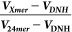	ferritin fragments
1200	100%	24-mer
1154	91%	22-mer
1024	68%	16-mer
943	53%	12-mer
890	44%	10-mer
810	29%	8-mer
730	16%	4-mer
611	7%	2-mer
645	0%	released

We note that the subunits always disassociate in single or multiple dimers, due to the well-known stable twofold axis of the ferritin. The 24 subunits of ferritin connect through hydrogen bonding and electrostatic interactions, forming two-, three-, and fourfold symmetries.^12,49,50^ Among these symmetries, the twofold axis is the most stable one due to its largest number of interaction sites. Conversely, the threefold axis is the weakest one because of its fewest interaction sites.^12,50^ Therefore, there is a large chance that the initial disassembly of ferritin occurs in one of the threefold channels.^12^

**Figure 5:**
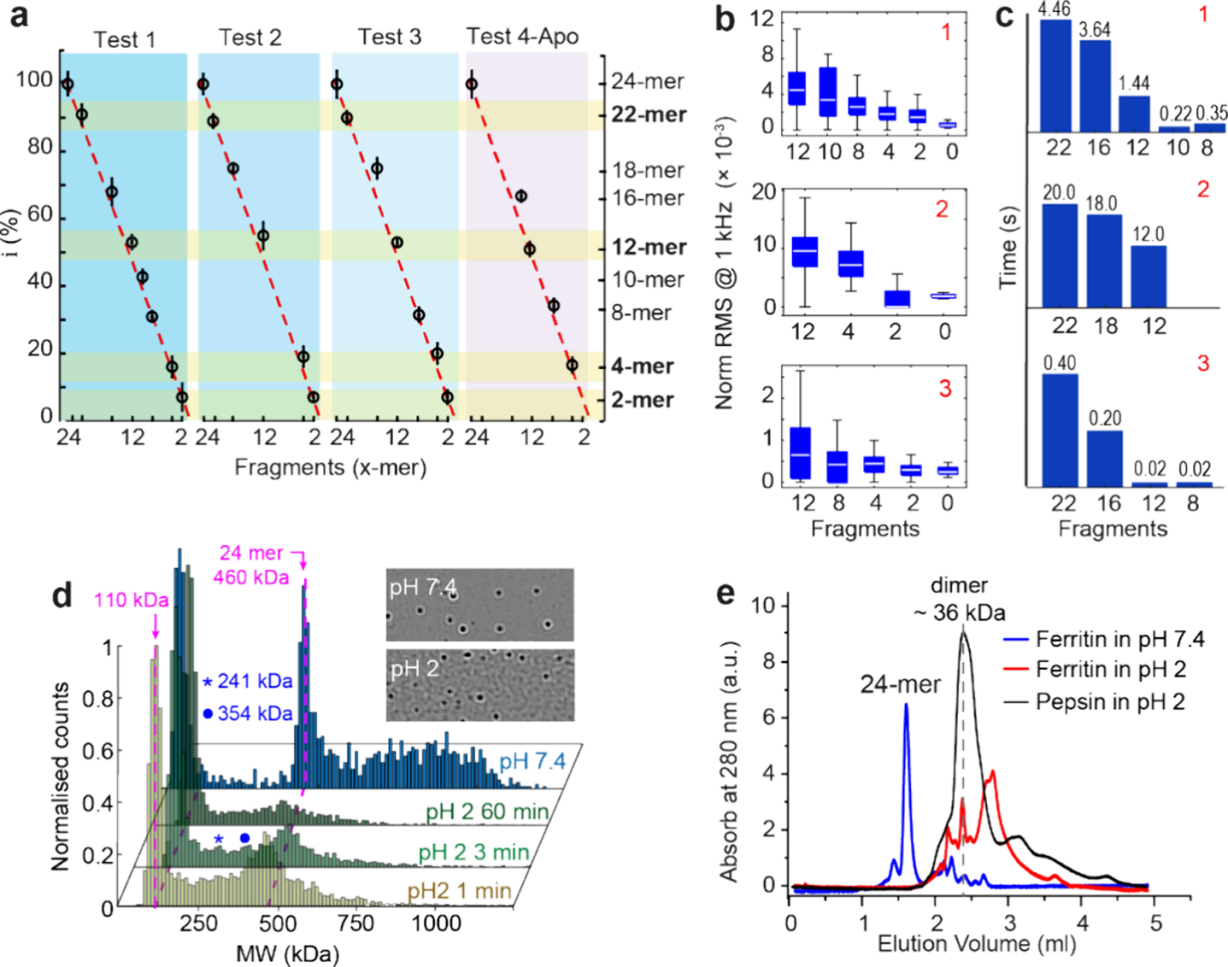
Single ferritin molecule disassembly and its kinetics in four tests. (a) Number of subunits remaining in the trap during disassembly for three tests of holo-ferritin (tests 1–3) and one test of apo-ferritin (test 4). The subunit numbers were calculated according to eq 5. The red dashed lines connect 24-mer (100%) and 0-mer (0%) of each data set. (b) Normalized RMS values of disassembly steps from 12-mer to protein released in the three tests related to holo-ferritin. (c) Dwelling time of the subunits in the trap for tests 1–3. Tetramer and dimer are excluded as a tetramer can disassociate into two dimers that coexist in the trap. (d) MP of ferritin at pH 2 across various time points (1, 3, and 60 min), along with ferritin at pH 7.4. The PDF is normalized to maximum counts for fair comparison among different measurements. The asterisk and circle mark the disappearance of intermediate subunits as ferritin is exposed to an acidic environment. Sample interferometric contrast images of binding events acquired from ferritin in pH 7.4 and pH 2. All images are on the same contrast scale with a field of view of 10.8 × 6.8 μm. (e) Size-exclusion chromatography of ferritin at pH 7.4 (blue) and pH 2 (red) and pepsin at pH 2 (black). See the peak positions of protein markers in pH 7.4 in Figure S11.

Among five disassembly experiments of a single ferritin molecule, except one experiment where the ferritin was released from the trap before the disassembly (Figure S7), four experiments exhibited a step-by-step disassembly signal. These experiments are denoted as tests 1, 2, 3, and 4, where test 1 corresponds to the findings depicted in Figure 4 and Table 1, and the disassembly traces of tests 2 and 3 can be found in Figures S8 and S9, respectively. In addition to holo-ferritin, test 4 presents the disassembly process of an apo-ferritin at pH 2, with a transmission trace shown in Figure S10. All four transmission traces exhibit distinct drops after ferritin is subjected to pH 2.0 followed by protein release as the signal decreases to the baseline level. Figure 5a identifies the number of remaining subunits based on the transmission level during the disassembly process, as described by eq 5, where the *V*_Xmer_ is the mean transmission level of each step during disassembly:
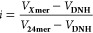
5

The high sensitivity of optical nanotweezers enables us to discover a detailed pathway of ferritin disassembly through different fragments (Figure 5a), which is otherwise difficult for other single-molecule techniques. We found that the disassembly of holo-ferritin always starts with the disassociation of a dimer, forming a 22-mer assembly structure within the trap. This 22-mer fragment can go through various fragments during the disassembly. Despite the difference in the fragmentation pathway across the three tests of holo-ferritin, certain subunits (22-mer, 12-mer, 4-mer, 2-mer) consistently maintain their integrity across the disassembly of holo-ferritin (test 1–3), as highlighted in Figure 5a. To the best of our knowledge, no other technique can provide such detailed insight into ferritin disassembly.

It is important to mention that the analysis presented above is based on the spherical approximation of the oligomers. An anisotropic shape can lead to a larger transmission change compared to a spherical shape.^28^ In Figure 5a, we introduced lines connecting 24-mer to 0-mer in each of the four data sets. Although most data points align closely with these lines, they exhibit slight deviations above the linear lines. This slightly higher transmission value may be attributed to the anisotropic shape of the fragments, resulting in a higher polarizability compared to that of a spherical particle.

The normalized RMS values presented in Figure 5b, spanning from 12-meric structures to the fragments released, agree with prior research that the RMS value of the transmission trace decreases with the size of the trapped globular proteins.^47^ To our surprise, the smaller fragment leaves the trap immediately after disassembly occurs. This fact is evidenced by the consistent RMS within each transmission level; if two fragments were coexisting inside the trapping site, their Brownian motion would lead to increased noise in the transmission trace compared to that of a single entity. We did not observe the coexistence of two fragments within the trapping site, except in the case of a tetramer (or two dimers), where the fragments have identical size hence leading to significant noise difference, as shown in the transmission trace in Figure 4a and Figure S9c.

The high RMS of the 12-mer fragment arises from its large accessible areas at the subunit–subunit interfaces, leading to a reduced number of hydrogen bonds between dimer–dimer interactions.^44^

Previous research on the disassembly and assembly kinetics of ferritin has employed techniques like circular dichroism with SAXS and native PAGE,^51−53^ but these methods primarily studied ferritin in bulk measurement. Disassembly kinetics at the single-molecule level have not been thoroughly investigated. The optical nanotweezer approach allows one to resolve the dwell time of each subunit during disassembly down to events occurring on the millisecond time scales (Figure 5c). We observed a cooperative disassembly, where the disassembly of each subunit facilitates and accelerates the disassembly of the entire structure. This cooperative disassembly process has been demonstrated by simulation previously.^54^ In the case of tetramer and dimer, the dwell time might not be accurate as tetramers can dissociate into two dimers and these two fragments can coexist in the trap as shown in Figure 4a.

We further validate our findings on single ferritin molecule fragmentation at pH 2 using MP as a complementary single-molecule approach, as demonstrated in Figure 5d. At pH 7.4, ferritin mainly existed at approximately 460 kDa, corresponding to its 24-mer fully assembled structure. We also observed small fragments at 60 kDa, possibly due to the impurity of the protein sample, which is consistent with the fast protein liquid chromatography (FPLC) result in Figure 5e (blue). At pH 2, the dominant peak shifted to a lower mass (∼110 kDa). We identified two intermediate masses in the ferritin sample at pH 2, potentially corresponding to 12-mer (asterisk) and 18-mer (circle). These intermediate fragments decrease with prolonged exposure of ferritin to pH 2, reinforcing our observation that ferritin undergoes a stepwise disassembly process. Although MP allowed us to comprehend the impact of acidic pH on the mass of ferritin fragments, it cannot provide details about their dynamic behavior and their dwelling time during the disassembly of each subunit. Additionally, at lower molecular weights, as depicted in Figure 5e, it did not reveal the tetrameric and dimeric states of ferritin due to the intrinsic technical limit of the instrument when operating at more extreme conditions like low pH. However, by using FPLC, we confirmed the dimeric state of ferritin at pH 2 (36 kDa), as it overlays well with the position of pepsin (Figure 5e), which is stable at pH 2 and has a molecular weight of 35 kDa. For further details about FPLC and MP in this work, see the Supporting Information, Figures S11 and S12.

## Conclusions

This study used DNH structures in an optical tweezer system to investigate the impact of different concentrations of AA and acidic pH on single ferritin molecules *in situ*. At the single-molecule level, we observed that ferritin’s dynamics gradually increased with AA concentration before reaching a saturation concentration (5 mM), suggesting a higher rate of Fe^3+^ to Fe^2+^ reduction in its core. When exposed to a highly acidic environment, i.e., pH 2, ferritin and apo-ferritin became highly dynamic due to protein swelling and hydrogen bonding protonation and eventually underwent a disassembly process. We identified the number of subunits during its disassembly pathway and discovered the most significant fragments consistently existing during the disassembly, which are 22-mer, 12-mer, 4-mer, and 2-mer. The 12-mer fragment is particularly significant as it shows the highest protein dynamics of all subunits. This study further provides the kinetics of single ferritin molecule disassembly under acidic pH conditions, revealing cooperative disassembly kinetics. Overall, these findings on the single-molecule level provide valuable insights into the potential engineering of ferritin’s structure to achieve desired functions like designing molecular machines and drug delivery platforms.^55,56^ Furthermore, this study showcases the quantitative analytical potential of optical nanotweezers in biomolecule analysis. This capability extends to diverse protein applications in future research and lays the groundwork for other single-molecule techniques, including solid-state nanopores, and AFM in investigating ferritin.^57,58^

## Methods

### Fabrication of DNH Structures

The DNH structures used in this study were fabricated by following the method outlined in previous works.^26,28^ Initially, 550 μm of thick fused silica wafers was coated with a 30 nm silicon nitride (SiN_*x*_) layer using low-pressure chemical vapor deposition (LPCVD) at 800 °C. Subsequently, a 5 nm adhesion layer of titanium (Ti) and a 100 nm layer of gold (Au) were deposited on the silicon nitride using electron beam evaporation (Leybold Optics LAB 600H) at 190 °C. The silica wafers were then diced into 10 mm × 10 mm chips using a dicing machine (Disco DAD321).

To create the DNH structures within the gold film, a focused ion beam (FIB) with a gallium ion source (Zeiss Crossbeam 550) was employed. The DNH geometry consisted of two circles with a diameter of 160 nm, spaced 200 nm apart, and connected by a 3 × 40 nm rectangle. The FIB parameters were optimized with an ion beam energy of 30 kV, a beam current of 1 pA, and dwell times of 1.25 μs for the circles and 5 μs for the rectangle. These parameters ensured the creation of DNH structures with gap sizes predominantly around 20 nm, devoid of any residual gold. In this study, we employed five distinct DNH structures (no. 1, no. 2, no. 3, no. 4, and no. 5), with each structure indicated in the corresponding figure captions. Additionally, Figure S1 displays SEM images of the DNH structures at a 20° tilt together with precise measurements of relevant dimensions of the holes and the gap between the holes.

### Optical Nanotweezer Setup

Optical components were obtained from Thorlabs, as previously described.^26^ The laser was focused on a spot of approximately 1.2 μm diameter using a 60× Plan Fluor objective (Nikon, Tokyo, Japan) with a numerical aperture (NA) of 0.85. A half-wave plate was used to adjust the laser’s polarization to be perpendicular to the line connecting the centers of two holes. The power density at the DNH sample was 19 mW/μm^2^, which resulted from the incident laser power of 32 mW before the objective. The transmitted light was collected by a silicon APD (APD120A, Thorlabs), which converted the optical intensity to a voltage signal. The APD’s voltage signal was recorded by a data acquisition card (USB-6361, NI) at a sampling rate of 1 MHz using a customized LabVIEW program.

### Preparation of Proteins, Different pH Buffers, and AA Solutions

#### Protein Solutions

We procured holo-ferritin (derived from equine spleen, F4503) and apo-ferritin (from equine spleen, A3660), along with other chemicals, from Sigma-Aldrich in the UK. For the trapping experiments, we employed 0.5 μM ferritin in 0.1 M phosphate buffer (PB) with a pH value of 7.4.

#### Different pH Solutions

To create solutions with varying pH levels, we adjusted the pH by adding hydrochloric acid (HCl) with a concentration of 0.1 M (purchased from Fisher Scientific, product code 15697310) to PB buffer (pH 7.4). Also, we conducted experiments in both a pH 7.4 phosphate buffer (PB) and a 0.1 M glycine–HCl buffer at pH 2.0 The glycine–HCl buffer served as a control to assess the effects of a sudden pH shift from 7.4 to 2.0.

#### AA-Buffer Solution

l-AA (A5960) was dissolved in PB buffer (pH 7.4) at three different concentrations (1, 5, and 10 mM) and was prepared freshly before each test.

### Fluidic System

The flow cells used in this study are identical to those previously reported.^26,28^ We produced the flow cells using a FormLab 2 printer employing Clear V4 resin with a resolution of 50 μm (Formlabs Inc., USA). Samples in the flow cell were sealed with a cover glass by using a two-component silicone glue (Twinsil, Picodent, Germany). The DNH sample and cover slide were separated by a 50 μm thick double-sided tape (ARcare92712, Adhesive Research, Inc.), creating a fluidic channel with a volume of 3.5 μL. A syringe pump (Harvard Apparatus, US) controlled the flow rate and direction through a 12-port valve (Mux Distrib, Elve Flow, France). To replace the buffer after protein trapping, we introduced buffer into the flow chamber at a flow rate of 3 μL/min. Considering the internal diameter of the tubing in the flow controller system and the mentioned flow rate, the different pH and AA solutions reach the flow chamber 25 μL (6 min after injection) after passing through the flow controller.

### Size Exclusion Chromatography

The pH-induced disassembly of ferritin was investigated through size exclusion chromatography (SEC) utilizing a Superose 6 Increase 3.2/300 column. Molecular sizes of both the intact ferritin assembly and its constituent fragments were determined under various conditions by comparing their elution volumes to those of standard proteins, namely, aprotinin (6.5 22 kDa, Sigma-Aldrich A3886), carbonic anhydrase (29 kDa, Sigma-Aldrich C7025), bovine serum albumin (66 kDa, Sigma-Aldrich A8531), Alcohol Dehydrogenase (150 kDa, Sigma-Aldrich A8656), beta-amylase (200 kDa, Sigma-Aldrich A8781), apo-ferritin (443 kDa Sigma- Aldrich A3660), and thyroglobulin (669 kDa, Sigma-Aldrich T9145). The concentration of all protein markers was maintained at 0.165 mg/mL, except for ferritin, which was at a concentration of 1.65 mg/mL.

To assess retention times and ensure the accurate positioning of the peak at pH 2, pepsin, a stable protein under acidic conditions with a molecular weight of 35 kDa, was used at a concentration of 0.4 mg/mL. The mobile phase composition consisted of 10 mM PB buffer with 50 mM sodium chloride, pH 7.4, and, for acidic conditions, 100 mM glycine–HCl at pH 2. All SEC experiments were conducted using the KTA-Explorer FPLC system with protein detection by absorption at 280 nm.

### Single-Molecule Mass Photometry Analysis

MP measurements were performed using a TwoMP mass photometer (Refeyn), adhering to the manufacturer’s calibration guidelines. Native Mark (Thermo Fisher Scientific) was employed for calibration in a T50 buffer at pH 7.4. The calibration accuracy was estimated to be 1.5% (*R*_2_ = 0.99997). The experiments were carried out using conventional microscope cover glasses (Marienfeld, no 1.5 H) cleaned by rinsing with deionized water (×5) and isopropanol (×5) followed by drying under a N_2_ flow, using a silicon gasket (Grace Biolabs) to confine the sample. For the entire measurement process, a total volume of 20 μL of mixed ferritin in buffers (50 nM) was used, which was then introduced into the pristine wells of the gaskets. To ensure procedural cleanliness, MP measurement buffers underwent filtration using 0.22 μm syringe filters. The protein adsorption events were obtained using AcquireMP software (Refeyn) after recording 60 s videos for each sample. Data analysis was performed using DiscoverMP software (Refeyn) and OriginPro 2021 (OriginLab).

### Data Analysis

In this study, MATLAB scripts were employed for the analysis of all of the presented data. Raw data underwent filtration using a zero-phase Gaussian low-pass filter with a specified cutoff frequency (1 or 5 kHz) applying the filtfilt.m function. Probability density functions (PDFs) were determined using the ksdensity.m function and filtered at 10 Hz. The normalized RMS values (Figures 2 and 3) were calculated by dividing the standard deviation of a 0.5-s trace by its mean value and are statistically shown as a boxplot for a 5-s trace at different time intervals.

The trace length for the last figure was 0.015 s due to the short duration of each step.

## Data Availability

The data that support the findings of this study are available upon reasonable request from the authors.

## Supporting Information Available

S1: scanning electron microscopy (SEM) image of the double nanohole (DNH); S2: optical properties of the DNH sample; S3: repeated testing of AA’s effect on single ferritin dynamics at various concentrations; S4: effect of AA on single apo-ferritin molecule (ferritin without iron core); S5: effect of high concentration of AA (1.5 M) on single ferritin; S6: transition time between different transmission levels; S7: nonstepwise disassembly of a single ferritin molecule upon exposure to pH 2; S8: stepwise disassembly trace of test 2; S9: stepwise disassembly trace of test 3; S10: stepwise disassembly trace of Apo-ferritin; S11: size exclusion chromatography (protein markers); S12: mass photometry (PDF)

## Notes

Arman Yousefi; Ze Zheng; Saaman Zargarbashi; Mahya Assadipapari; Graham J Hickman; Christopher DJ Parmenter; Carlos J Bueno-Alejo; Gabriel Sanderson; Dominic Craske; Lei Xu; Mohsen Rahmani; Cuifeng Ying; Structural Flexibility and Disassembly Kinetics of Single Ferritins Using Optical Nanotweezers. 2023, 2023.09.22.558948, BioRxiv, doi: 10.1101/2023.09.22.558948 (accessed January 25, 2024).

## References

[ref1] Liu X.; Theil E. C. Ferritins: Dynamic Management of Biological Iron and Oxygen Chemistry. Acc. Chem. Res. 2005, 38 (3), 167–175. 10.1021/ar0302336. 15766235

[ref2] Moore P. G.; Rainbow P. S. Ferritin Crystals in the Gut Caeca of Stegocephaloides Christianiensis Boeck and Other Stegocephalidae (Amphipoda Gammaridea): A Functional Interpretation. Philos. Trans. R. Soc. B 1984, 306 (1127), 219–245. 10.1098/rstb.1984.0086.

[ref3] Gonciarz R. L.; Collisson E. A.; Renslo A. R. Ferrous Iron-Dependent Pharmacology. Trends Pharmacol. Sci. 2021, 42 (1), 7–18. 10.1016/j.tips.2020.11.003. 33261861 PMC7754709

[ref4] Bresgen N.; Eckl P. M. Oxidative Stress and the Homeodynamics of Iron Metabolism. Biomolecules. 2015, 5 (2), 808–847. 10.3390/biom5020808. 25970586 PMC4496698

[ref5] Sana B.; Johnson E.; Lim S. The Unique Self-Assembly/Disassembly Property of Archaeoglobus Fulgidus Ferritin and Its Implications on Molecular Release from the Protein Cage. Biochim Biophys Acta Gen Subj 2015, 1850 (12), 2544–2551. 10.1016/j.bbagen.2015.08.019. 26341788

[ref6] Takahashi T.; Kuyucak S. Functional Properties of Threefold and Fourfold Channels in Ferritin Deduced from Electrostatic Calculations. Biophysical journal 2003, 84 (4), 2256–2263. 10.1016/S0006-3495(03)75031-0. 12668434 PMC1302792

[ref7] Bridges K. R.; Hoffman K. E. The Effects of Ascorbic Acid on the Intracellular Metabolism of Iron and Ferritin. J. Biol. Chem. 1986, 261 (30), 14273–14277. 10.1016/S0021-9258(18)67014-0. 3464594

[ref8] Brissot P.; Deugnier Y.; Le Treut A.; Regnouard F.; Simon M.; Bourel M. Ascorbic Acid Status in Idiopathic Hemochromatosis. Digestion 1978, 17 (6), 479–487. 10.1159/000198154. 710734

[ref9] O’Brien R. T. Ascorbic acid enhancement of desferrioxamine-induced urinary iron excretion in thalassemia major. Ann. N.Y. Acad. Sci. 1974, 232 (1), 221–225. 10.1111/j.1749-6632.1974.tb20588.x. 4528431

[ref10] Zhang P.; Omaye S. T. β-Carotene and Protein Oxidation: Effects of Ascorbic Acid and α-Tocopherol. Toxicology 2000, 146 (1), 37–47. 10.1016/S0300-483X(00)00160-8. 10773361

[ref11] Harrison P. M. The Structure and Function of Ferritin. Biochem. Educ. 1986, 14 (4), 154–162. 10.1016/0307-4412(86)90203-7.

[ref12] Kim M.; Rho Y.; Jin K. S.; Ahn B.; Jung S.; Kim H.; Ree M. PH-Dependent Structures of Ferritin and Apoferritin in Solution: Disassembly and Reassembly. Biomacromolecules 2011, 12 (5), 1629–1640. 10.1021/bm200026v. 21446722

[ref13] Palombarini F.; Di Fabio E.; Boffi A.; Macone A.; Bonamore A. Ferritin Nanocages for Protein Delivery to Tumor Cells. Molecules 2020, 25 (4), 825 10.3390/molecules25040825. 32070033 PMC7070480

[ref14] Palombarini F.; Masciarelli S.; Incocciati A.; Liccardo F.; Di Fabio E.; Iazzetti A.; Fabrizi G.; Fazi F.; Macone A.; Bonamore A.; Boffi A. Self-Assembling Ferritin-Dendrimer Nanoparticles for Targeted Delivery of Nucleic Acids to Myeloid Leukemia Cells. J. Nanobiotechnology 2021, 19 (1), 172 10.1186/s12951-021-00921-5. 34107976 PMC8190868

[ref15] Valero E.; Fiorini S.; Tambalo S.; Busquier H.; Callejas-Fernández J.; Marzola P.; Gálvez N.; Domínguez-Vera J. M. In Vivo Long-Term Magnetic Resonance Imaging Activity of Ferritin-Based Magnetic Nanoparticles versus a Standard Contrast Agent. J. Med. Chem. 2014, 57 (13), 5686–5692. 10.1021/jm5004446. 24901375

[ref16] Fan K.; Gao L.; Yan X. Human Ferritin for Tumor Detection and Therapy. Wiley Interdiscip Rev. Nanomed Nanobiotechnol 2013, 5 (4), 287–298. 10.1002/wnan.1221. 23606622

[ref17] Song N.; Zhang J.; Zhai J.; Hong J.; Yuan C.; Liang M. Ferritin: A Multifunctional Nanoplatform for Biological Detection, Imaging Diagnosis, and Drug Delivery. Acc. Chem. Res. 2021, 54 (17), 3313–3325. 10.1021/acs.accounts.1c00267. 34415728

[ref18] Smith-Johannsen H.; Drysdale J. W. Reversible Dissociation of Ferritin and Its Subunits in Vitro. BBA - Protein Structure 1969, 194 (1), 43–49. 10.1016/0005-2795(69)90177-9. 4188449

[ref19] Stefanini S.; Vecchini P.; Chiancone E. On the Mechanism of Horse Spleen Apoferritin Assembly: A Sedimentation Velocity and Circular Dichroism Study. Biochemistry 1987, 26 (7), 1831–1837. 10.1021/bi00381a007. 3593696

[ref20] Harrison P. M.; Gregory D. W. Reassembly of Apoferritin Molecules from Subunits. Nature 1968, 220 (5167), 578–580. 10.1038/220578a0. 5686729

[ref21] Benni I.; Trabuco M. C.; Di Stasio E.; Arcovito A.; Boffi A.; Malatesta F.; Bonamore A.; De Panfilis S.; De Turris V.; Baiocco P. Excimer Based Fluorescent Pyrene-Ferritin Conjugate for Protein Oligomerization Studies and Imaging in Living Cells. RSC Adv. 2018, 8 (23), 12815–12822. 10.1039/C8RA00210J. 35541244 PMC9079363

[ref22] Platnich C. M.; Rizzuto F. J.; Cosa G.; Sleiman H. F. Single-Molecule Methods in Structural DNA Nanotechnology. Chemical Society Reviews. 2020, 49 (13), 4220–4233. 10.1039/C9CS00776H. 32538403

[ref23] Maity B.; Li Z.; Niwase K.; Ganser C.; Furuta T.; Uchihashi T.; Lu D.; Ueno T. Single-Molecule Level Dynamic Observation of Disassembly of the Apo-Ferritin Cage in Solution. Phys. Chem. Chem. Phys. 2020, 22 (33), 18562–18572. 10.1039/D0CP02069A. 32785391

[ref24] Mandal S. S. Force Spectroscopy on Single Molecules of Life. ACS Omega 2020, 5 (20), 11271–11278. 10.1021/acsomega.0c00814. 32478214 PMC7254507

[ref25] Adcock S. A.; McCammon J. A. Molecular Dynamics: Survey of Methods for Simulating the Activity of Proteins. Chem. Rev. 2006, 106 (5), 1589–1615. 10.1021/cr040426m. 16683746 PMC2547409

[ref26] Yousefi A.; Ying C.; Parmenter C. D. J.; Assadipapari M.; Sanderson G.; Zheng Z.; Xu L.; Zargarbashi S.; Hickman G. J.; Cousins R. B.; Mellor C. J.; Mayer M.; Rahmani M. Optical Monitoring of In Situ Iron Loading into Single. Native Ferritin Proteins. Nano Lett. 2023, 23 (8), 3251–3258. 10.1021/acs.nanolett.3c00042. 37053043 PMC10141409

[ref27] Young G.; Hundt N.; Cole D.; Fineberg A.; Andrecka J.; Tyler A.; Olerinyova A.; Ansari A.; Marklund E. G.; Collier M. P.; Chandler S. A.; Tkachenko O.; Allen J.; Crispin M.; Billington N.; Takagi Y.; Sellers J. R.; Eichmann C.; Selenko P.; Frey L.; Riek R.; Galpin M. R.; Struwe W. B.; Benesch J. L. P.; Kukura P. Quantitative Mass Imaging of Single Biological Macromolecules. Science (1979) 2018, 360 (6387), 423–427. 10.1126/science.aar5839. PMC6103225 29700264

[ref28] Ying C.; Karakaçi E.; Bermúdez-Ureña E.; Ianiro A.; Foster C.; Awasthi S.; Guha A.; Bryan L.; List J.; Balog S.; Acuna G. P.; Gordon R.; Mayer M. Watching Single Unmodified Enzymes at Work. arXiv 2021, arXiv-2107 10.48550/arXiv.2107.06407.

[ref29] Juan M. L.; Gordon R.; Pang Y.; Eftekhari F.; Quidant R. Self-Induced Back-Action Optical Trapping of Dielectric Nanoparticles. Nat. Phys. 2009, 5 (12), 915–919. 10.1038/nphys1422.

[ref30] Pang Y.; Gordon R. Optical Trapping of a Single Protein. Nano Lett. 2012, 12 (1), 402–406. 10.1021/nl203719v. 22171921

[ref31] Zheng Y.; Wu Z.; Ping Shum P.; Xu Z.; Keiser G.; Humbert G.; Zhang H.; Zeng S.; Quyen Dinh X. Sensing and Lasing Applications of Whispering Gallery Mode Microresonators. Opto-Electronic Advances 2018, 1 (9), 18001501–18001510. 10.29026/oea.2018.180015.

[ref32] Li P.; Chen Y.; Wang B.; Li M.; Xiang D.; Jia C.; Guo X. Single-Molecule Optoelectronic Devices: Physical Mechanism and Beyond. Opto-Electronic Advances. Chinese Academy of Sciences 2022, 5 (5), 210094–1. 10.29026/oea.2022.210094.

[ref33] Kotnala A.; DePaoli D.; Gordon R. Sensing Nanoparticles Using a Double Nanohole Optical Trap. Lab Chip 2013, 13 (20), 4142–4146. 10.1039/c3lc50772f. 23969596

[ref34] Zehtabi-Oskuie A.; Bergeron J. G.; Gordon R. Flow-Dependent Double-Nanohole Optical Trapping of 20 Nm Polystyrene Nanospheres. Sci. Rep 2012, 2 (1), 966 10.1038/srep00966. 23236587 PMC3520027

[ref35] Pang Y.; Gordon R. Optical Trapping of 12 Nm Dielectric Spheres Using Double-Nanoholes in a Gold Film. Nano Lett. 2011, 11 (9), 3763–3767. 10.1021/nl201807z. 21838243

[ref36] Peters M.; McIntosh D.; Branzan Albu A.; Ying C.; Gordon R. Label-Free Tracking of Proteins through Plasmon-Enhanced Interference. ACS Nanoscience Au 2024, 4 (1), 69–75. 10.1021/acsnanoscienceau.3c00045. 38406310 PMC10885339

[ref37] Timoshnikov V. A.; Kobzeva T. V.; Polyakov N. E.; Kontoghiorghes G. J. Redox Interactions of Vitamin c and Iron: Inhibition of the pro-Oxidant Activity by Deferiprone. Int. J. Mol. Sci. 2020, 21 (11), 3967 10.3390/ijms21113967. 32486511 PMC7312906

[ref38] Boyer R. F.; McCleary C. J. Superoxide Ion as a Primary Reductant in Ascorbate-Mediated Ferretin Iron Release. Free Radical Biol. Med. 1987, 3 (6), 389–395. 10.1016/0891-5849(87)90017-7. 2828195

[ref39] Badu-Boateng C.; Naftalin R. J. Ascorbate and Ferritin Interactions: Consequences for Iron Release in Vitro and in Vivo and Implications for Inflammation. Free Radical Biol. Med. 2019, 133, 75–87. 10.1016/j.freeradbiomed.2018.09.041. 30268889

[ref40] Jones T.; Spencer R.; Walsh C. Mechanism and Kinetics of Iron Release from Ferritin by Dihydroflavins and Dihydroflavin Analogues. Biochemistry 1978, 17 (19), 4011–4017. 10.1021/bi00612a021. 708692

[ref41] Badu-Boateng C.; Pardalaki S.; Wolf C.; Lajnef S.; Peyrot F.; Naftalin R. J. Labile Iron Potentiates Ascorbate-Dependent Reduction and Mobilization of Ferritin Iron. Free Radic Biol. Med. 2017, 108, 94–109. 10.1016/j.freeradbiomed.2017.03.015. 28336129

[ref42] Takeda S.; Yamaki M.; Ebina S.; Nagayama K. Site-Specific Reactivities of Cysteine Residues in Horse L-Apoferritin. J. Biochem 1995, 117 (2), 267–270. 10.1093/jb/117.2.267. 7608110

[ref43] Yoshizawa K.; Mishima Y.; Park S. Y.; Heddle J. G.; Tame J. R. H.; Iwahori K.; Kobayashi M.; Yamashita I. Effect of N-Terminal Residues on the Structural Stability of Recombinant Horse L-Chain Apoferritin in an Acidic Environment. J. Biochem 2007, 142 (6), 707–713. 10.1093/jb/mvm187. 17938140

[ref44] Li Z.; Maity B.; Hishikawa Y.; Ueno T.; Lu D. Importance of the Subunit–Subunit Interface in Ferritin Disassembly: A Molecular Dynamics Study. Langmuir 2022, 38 (3), 1106–1113. 10.1021/acs.langmuir.1c02753. 35015545

[ref45] Watt G. D.; Frankel R. B.; Papaefthymiou G. C. Reduction of Mammalian Ferritin. Proc. Natl. Acad. Sci. U. S. A. 1985, 82 (11), 3640–3643. 10.1073/pnas.82.11.3640. 3858840 PMC397841

[ref46] Theil E. C.; Liu X. S.; Tosha T. Gated Pores in the Ferritin Protein Nanocage. Inorg. Chim. Acta 2008, 361 (4), 868–874. 10.1016/j.ica.2007.08.025. PMC2350241 19262678

[ref47] Wheaton S.; Gordon R. Molecular Weight Characterization of Single Globular Proteins Using Optical Nanotweezers. Analyst 2015, 140 (14), 4799–4803. 10.1039/C5AN00026B. 25739349

[ref48] Quinten M. Optical Properties of Nanoparticle Systems: Mie and Beyond; 2010. John Wiley & Sons. 10.1002/9783527633135.

[ref49] Vedula L. S.; Brannigan G.; Economou N. J.; Xi J.; Hall M. A.; Liu R.; Rossi M. J.; Dailey W. P.; Grasty K. C.; Klein M. L.; Eckenhoff R. G.; Loll P. J. A Unitary Anesthetic Binding Site at High Resolution. J. Biol. Chem. 2009, 284 (36), 24176–24184. 10.1074/jbc.M109.017814. 19605349 PMC2782011

[ref50] Lawson D. M.; Artymiuk P. J.; Yewdall S. J.; Smith J. M. A.; Livingstone J. C.; Treffry A.; Luzzago A.; Levi S.; Arosio P.; Cesareni G.; Thomas C. D.; Shaw W. V.; Harrison P. M. Solving the Structure of Human H Ferritin by Genetically Engineering Intermolecular Crystal Contacts. Nature 1991, 349 (6309), 541–544. 10.1038/349541a0. 1992356

[ref51] Mohanty A.; Mithra K.; Jena S. S.; Behera R. K. Kinetics of Ferritin Self-Assembly by Laser Light Scattering: Impact of Subunit Concentration, PH, and Ionic Strength. Biomacromolecules 2021, 22 (4), 1389–1398. 10.1021/acs.biomac.0c01562. 33720694

[ref52] Sato D.; Ohtomo H.; Yamada Y.; Hikima T.; Kurobe A.; Fujiwara K.; Ikeguchi M. Ferritin Assembly Revisited: A Time-Resolved Small-Angle X-Ray Scattering Study. Biochemistry 2016, 55 (2), 287–293. 10.1021/acs.biochem.5b01152. 26690025

[ref53] Krausova K.; Charousova M.; Kratochvil Z.; Takacsova P.; Tesarova B.; Sivak L.; Peskova M. K.; Sukupova M.; Zivotska H.; Makovicky P.; Yamashita I.; Okamoto N.; Hynek D.; Haddad Y.; Pekarik V.; Rex S.; Heger Z. Toward Understanding the Kinetics of Disassembly of Ferritins of Varying Origin and Subunit Composition. Appl. Mater. Today 2022, 28, 101535 10.1016/j.apmt.2022.101535.

[ref54] Hilser V. J.; Dowdy D.; Oas T. G.; Freire E. The Structural Distribution of Cooperative Interactions in Proteins: Analysis of the Native State Ensemble. Proc. Natl. Acad. Sci. U. S. A. 1998, 95 (17), 9903–9908. 10.1073/pnas.95.17.9903. 9707573 PMC21434

[ref55] King N. P.; Sheffler W.; Sawaya M. R.; Vollmar B. S.; Sumida J. P.; André I.; Gonen T.; Yeates T. O.; Baker D. Computational Design of Self-Assembling Protein Nanomaterials with Atomic Level Accuracy. Science (1979) 2012, 336 (6085), 1171–1174. 10.1126/science.1219364. PMC4138882 22654060

[ref56] Chen H.; Zhang S.; Xu C.; Zhao G. Engineering Protein Interfaces Yields Ferritin Disassembly and Reassembly under Benign Experimental Conditions. Chem. Commun. 2016, 52 (46), 7402–7405. 10.1039/C6CC03108K. 27194454

[ref57] Yin Y.-D.; Chen F.-F.; Hu J.; Yang L.; Song X.-T.; Wu G.-R.; Xu M.; Gu Z.-Y. Solid-State Nanopore Distinguishes Ferritin and Apo-Ferritin with Identical Exteriors through Amplified Flexibility at Single-Molecule Level. Anal. Chem. 2023, 95 (45), 16496–16504. 10.1021/acs.analchem.3c02041. 37916987

[ref58] Nirmalraj P. N.; Rossell M. D.; Dachraoui W.; Thompson D.; Mayer M. In Situ Observation of Chemically Induced Protein Denaturation at Solvated Interfaces. ACS Appl. Mater. Interfaces 2023, 15 (41), 48015–48026. 10.1021/acsami.3c10510. 37797325 PMC10591235

