# Supplementary material for: Structural Flexibility and Disassembly Kinetics of Single Ferritin Molecules Using Optical Nanotweezers
Source: ACS Nano. 2024 Jun 8;18(24):15617–26. doi: 10.1021/acsnano.4c01221 (PMC11191739; doi:10.1021/acsnano.4c01221)
Supplement: Supplementary file 1 — nn4c01221_si_001.pdf [file nn4c01221_si_001.pdf]

Supporting Information:

Structural Flexibility and Disassembly Kinetics of Single Ferritin Molecules Using Optical Nanotweezers

Authors: Arman Yousefi[1], Ze Zheng[1], Saaman Zargarbashi[1], Mahya Assadipapari[1], Graham J. Hickman[2], Christopher D. J. Parmenter[3], Carlos J. Bueno-Alejo[4], Gabriel Sanderson[1], Dominic Craske[2], Lei Xu[1], Carole C. Perry[5], Mohsen Rahmani[1], and Cuifeng Ying[1*]*

[1] Advanced Optics and Photonics Laboratory, Department of Engineering, School of Science and Technology, Nottingham Trent University, Nottingham NG118NS, United Kingdom

[2] School of Science and Technology, Nottingham Trent University, Nottingham NG11 8NS, United Kingdom

[3] Nanoscale and Microscale Research Centre, University of Nottingham, Nottingham NG7 2RD, United Kingdom

[4]School of Chemistry, University of Leicester, University Road, Leicester LE1 7RH, United Kingdom

[5]Interdisciplinary Biomedical Research Centre, School of Science and Technology, Nottingham Trent University, Nottingham NG11 8NS, United Kingdom

Corresponding Authors Email: cuifeng.ying@ntu.ac.uk; mohsen.rahmani@ntu.ac.uk

S1- Scanning electron microscopy (SEM) image of the double nanohole (DNH).

In this study, we utilised five distinct DNH nanostructure designs, each specified in Figure S1. Despite employing identical FIB parameters as outlined in the materials and methods section, variations in gap size were observed. As previously investigated in our research, these differences in gap size can potentially impact the transmission signal.[1]

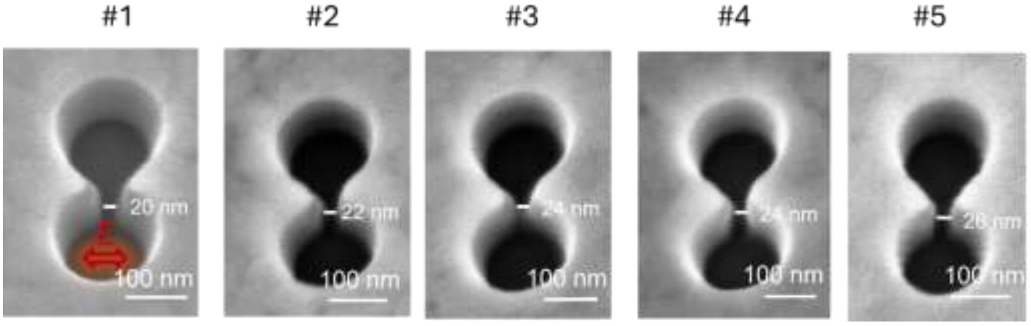

Figure S1. Scanning electron microscopy (SEM) images of the DNH structures with a 20° tilted angle were used in this work. Five DNH structures are utilised in this paper, with the polarisation direction indicated in #1. The DNH structures used in each figure are specified in the figure captions.

S2- Optical properties of the DNH sample

The trapping mechanism in this system relies on self-induced back action (SIBA) trapping, which offers the advantage of not being strictly limited to a specific excitation wavelength.[2] Figure 1b in the main text shows that DNH provides a highly confined field enhancement in the gap, enabling a strong gradient force for the efficient trapping of single proteins.

Finite Element Simulation: The field distribution of DNH was simulated using the finite element method (FEM) by COMSOL Multiphysics 6.2. We considered a material stack consisting of a 100 nm Au layer, 5 nm of Ti, and 30 nm of SiN_x on a fused silica substrate, surrounded by water with a refractive index (RI) of 1.333. Based on the SEM image in Figure S1 we analysed the gap geometry which exhibits a trapezium shape. The gap size, corresponds to a trapezium with a smaller base of 20 nm, as illustrated in Figure 1b, y-z plane in the main text. To account for tapering in the DNH structures, we introduced an 'n side truncated pyramid' structure with an RI of 1.333, transitioning from ~20 nm gap width at the bottom to ~45 nm at the top of the Au layer.

Linear Relationship between transmission change and particle volume: Spherical particles (with refractive index of 1.8) with radii ranging from 1 to 8 nm in 1nm intervals were positioned at the bottom of the DNH gap (Figure S2a). A plane-wave incidence along the z-axis at 852 nm was used as the light source, with the polarisation indicated by red arrows in Figure S2a. We consider the transmission value without the particle as the baseline (T_0). The relative transmission difference was calculated as $(T\text{-}T_0)/T_0$, where T is the transmission value for different spheres. Figure S2b demonstrates a clear linear correlation between these parameters.

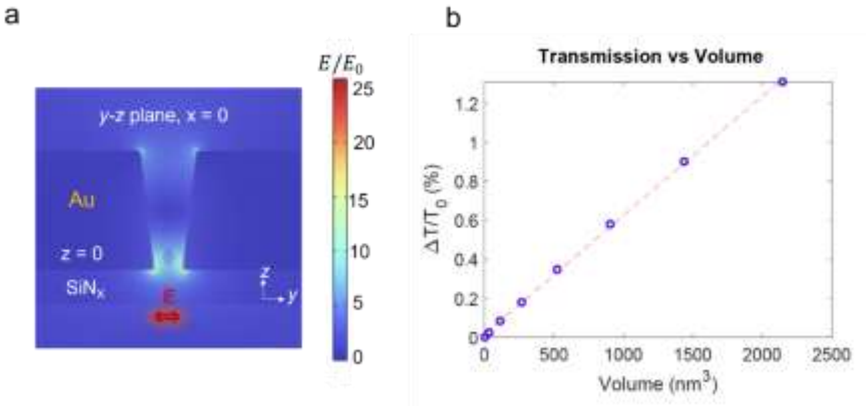

Figure S2. Simulated relationship between particle size and transmission. (a) Simulated distribution of electric field enhancement in the DNH structure in the y-z plane when a spherical particle with refractive index of 1.8 and radius of 6 nm is in the trap of DNH. (b) Depicting the relationship between the particle size (from 1 nm to 8 nm radius) and transmission signal in the hotspot of DNH.

S3- Repeat testing of the effect of ascorbic acid on single ferritin dynamics at various concentrations.

In addition to the results depicted in Figure 2 of the main text, we conducted replicates of the experiment (Figure S3). Remarkably, our repeated trials yielded findings that closely resembled those initially observed.

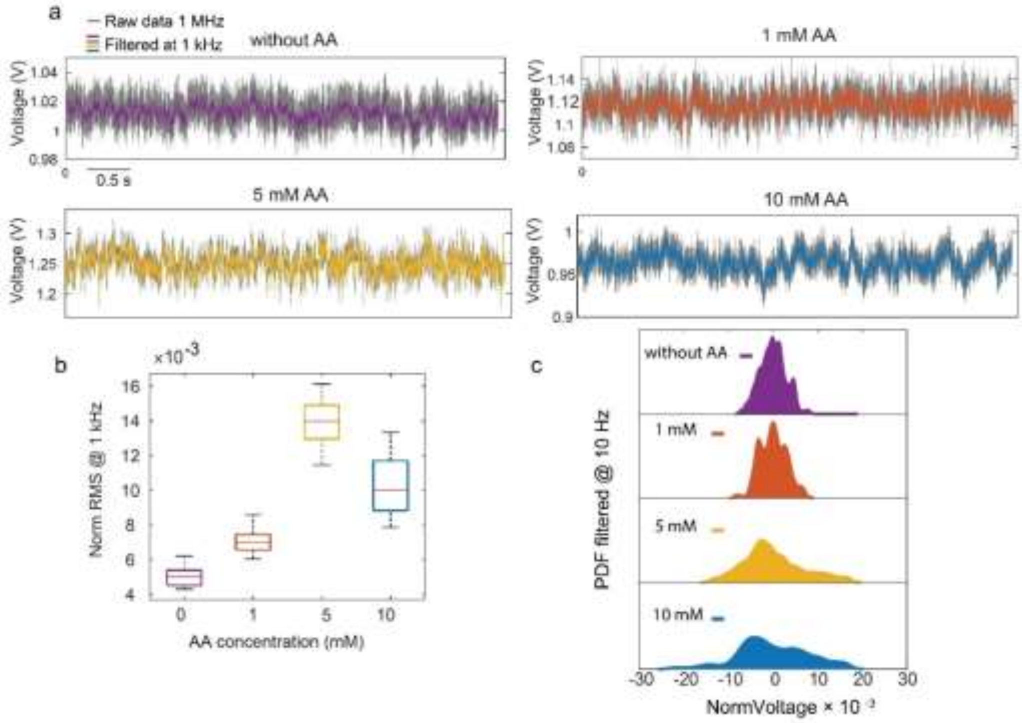

Figure S3. (a) Transmission signal of trapped ferritin within DNH (structure #2 – see Figure S1) at different concentrations of ascorbic acid (0 mM, 1 mM, 5 mM, and 10 mM). (b) Normalised root-mean-square (RMS) of 5-second transmission signal when ferritin is trapped in PB buffer and introduced to different ascorbic acid concentrations. The data were Gaussian-filtered at 1 kHz and collected every 0.5

seconds; the box plot displays the 25th and 75th percentiles of the data (c) PDF of 5-second traces of single ferritin molecule and different ascorbic acid concentrations (filtered at 10 Hz). Data were acquired at 1 MHz and then Gaussian filtered at 1 kHz.

S4- Effect of ascorbic acid on single apo-ferritin molecule (ferritin without iron core).

Figure S4 illustrates the trapping of a single apo-ferritin molecule in the absence of ascorbic acid and subsequent exposure to 5 mM ascorbic acid. We observed an increase in the RMS of the transmission when exposed to ascorbic acid, indicating ferritin channel opening and closing. We attribute this activity to the interaction between electron donors, such as superoxide radicals (O2•−) or ascorbate anions (AscH-), [3] or ascorbate radicals Asc•- (AFR), with the residues in the ferritin channels.[4] Figure S4 confirms that this interaction can occur in the channels even in the absence of a Fe^{3+} containing core.

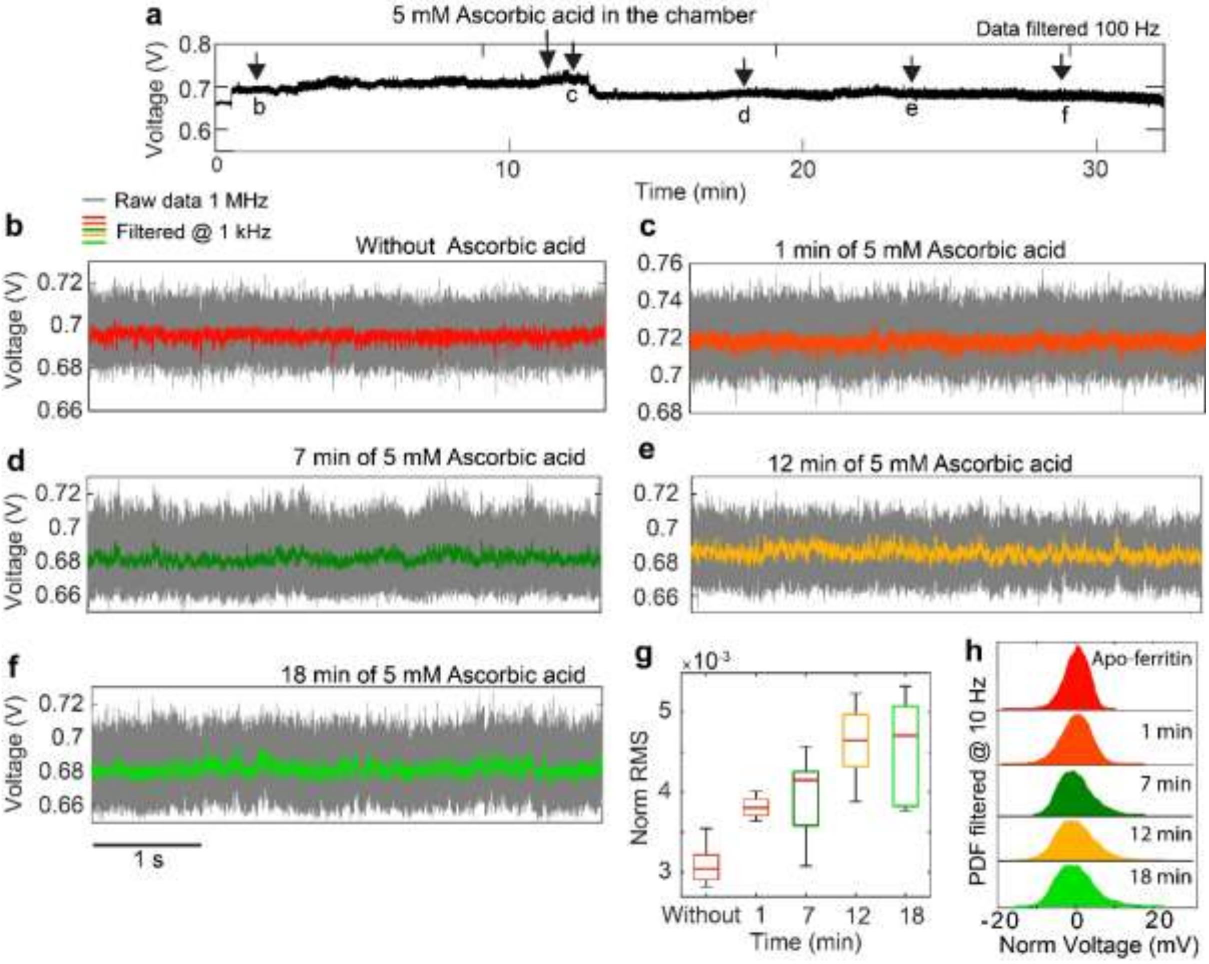

Figure S4. Effect of ascorbic acid on a single apo-ferritin molecule. (a) Continuous transmission signal of a single apo-ferritin trapped in the hotspot of a DNH (structure #5 – see SEM image in Figure S1) in PB buffer and exposed to 5 mM of ascorbic acid. (b-f) 5-second transmission traces of trapped apo-ferritin taken at different times indicated in panel a. (g) Normalised RMS of the transmission traces when ferritin is trapped in PB buffer and introduced to 5 mM ascorbic acid for different durations. The RMS values were calculated for every 0.5 s from the traces after digitally filtering at 1 kHz, as shown

in panel b-f. The box plot displays the 25th and 75th percentiles of the data. (h) PDF of 5-second transmission traces in panel b-f after filtering at 10 Hz. Raw data was collected at 1MHz.

S5 - Effect of high concentration of ascorbic acid (1.5 M) on a single ferritin molecule.

Elevated concentrations of ascorbic acid, approximately 1.5 M, significantly lower the buffer pH to around pH 2. As illustrated in Figure S5, exposure to ascorbic acid for approximately 12 minutes results in a notable drop in the transmission signal, accompanied by protein disassembly (as evident in the enlarged trace in Figure S5a). Figures S5b and S5c depict transmission signals collected before and after exposure to 1.5 M ascorbic acid, respectively. Analysis of their normalised RMS values reveals an increase post-exposure, indicating heightened dynamics induced by the high concentration of ascorbic acid (Figure S5d).

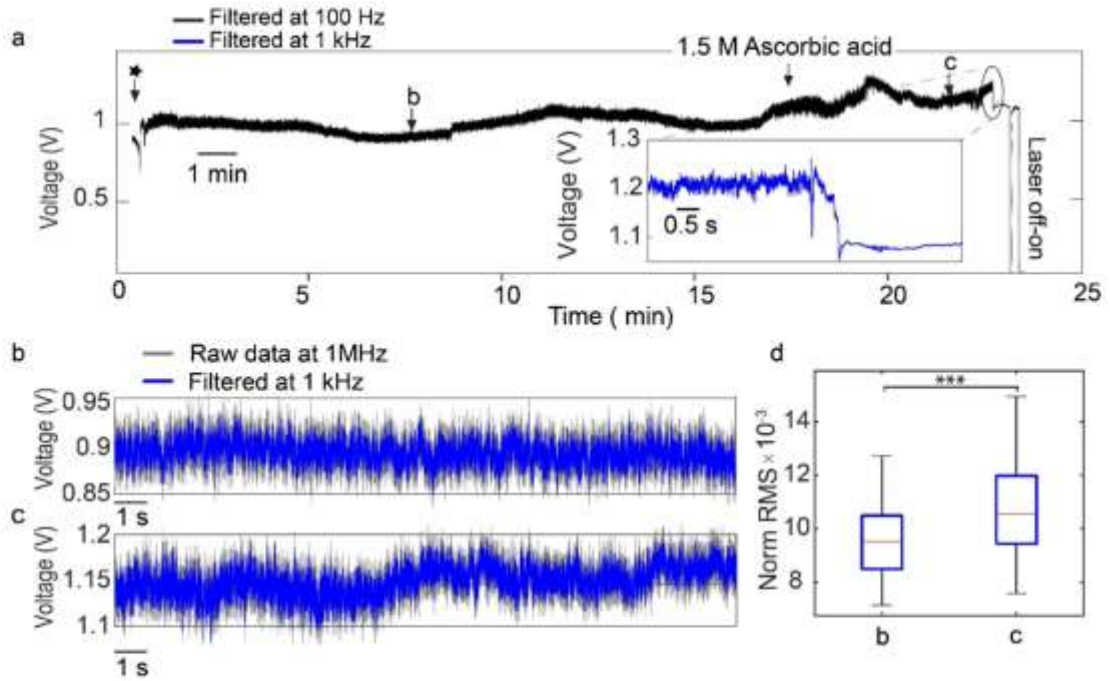

Figure S5. Disassembly of a single ferritin molecule exposed to 1.5 M ascorbic acid using DNH (structure #2 – see Figure S1). (a) The whole transmission trace depicts the protein trapping (indicated by the star), exposure to 1.5 M ascorbic acid (arrow), disassembly (magnified in the inset), and release by turning off the laser. (b) 20-s magnified transmission signal from panel a (marked by the arrow). (c) 20-s magnified transmission signal from panel a during exposure to 1.5 M ascorbic acid. (d) Boxplot of normalised RMS for 20-second transmission traces shown in panels b and c, collected at 0.5-second intervals at 1 kHz frequency. Asterisks indicate statistically significant differences (***$p < 0.0015$).

S6 – Transition time between different transmission levels.

Figure S6 provides zoomed-in views of the transitions between different steps shown in Figure 4. The transitions occur within a timescale ranging from 12 ms to 70 ms. This transition time, however, represents a combination of dissociation and the departure of the smaller fragment from the trapping site, rather than solely dissociation dynamics.

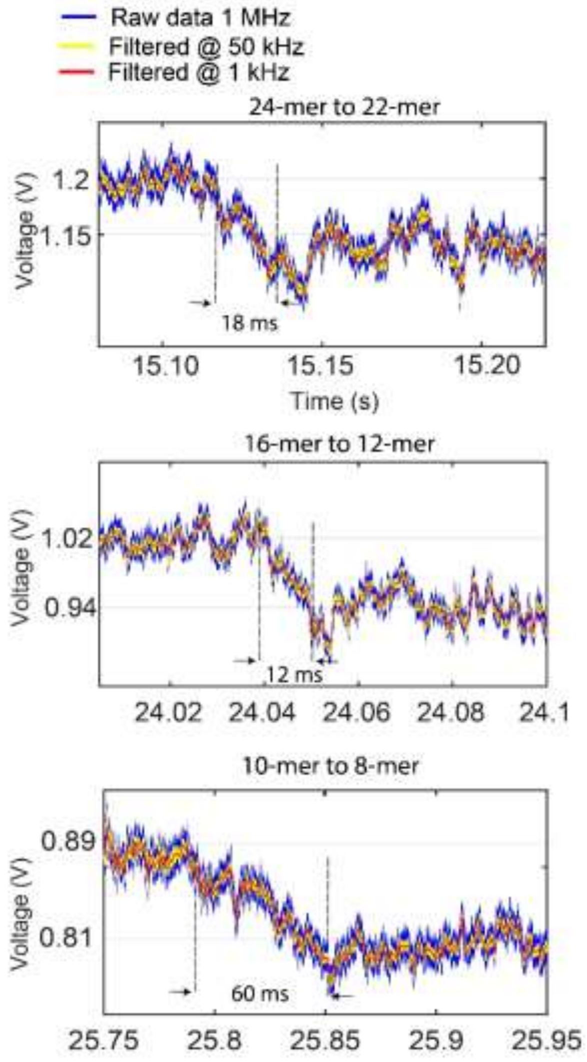

Figure S6. Zoomed-in views of the disassembly trace of single ferritin molecule upon exposure to pH 2, (disassembly trace in Figure 4). The grey lines represent the mean value of transmission levels listed in Table 1. The dashed lines mark the duration between the moment when the transmission signal (filtered at 1 kHz) departs from the upper level and when it arrives at the lower level. Data are shown as raw (blue), digital filtered at 50 kHz (yellow), and 1 kHz (red).

S7 – Non-stepwise disassembly of single ferritin molecule upon exposure to pH 2.

During repeated experiments exposing single ferritin molecule to pH 2, we consistently observed disassembly events, along with instances of sudden protein fragmentation.

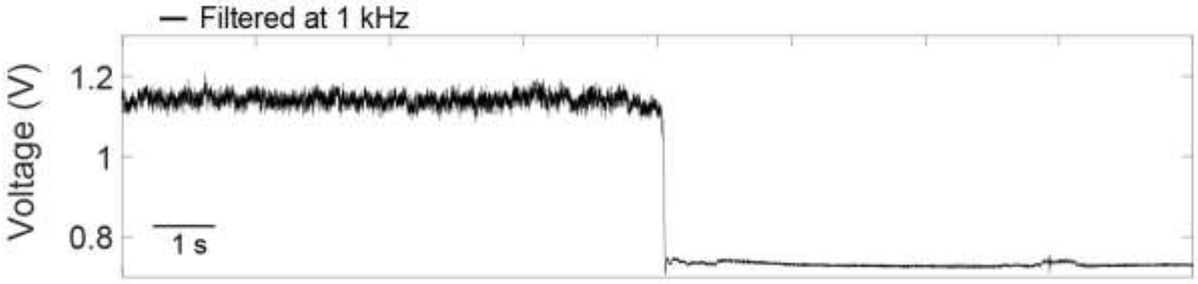

Figure S7. A trace of single ferritin molecule disassembly upon exposure to pH 2 showing non-stepwise behaviour. Data acquired at 1 MHz, in this figure, 1 kHz filtered data is showcased. (DNH structure #2 used for this test– see Figure S1)

S8 – Stepwise disassembly trace of test 2

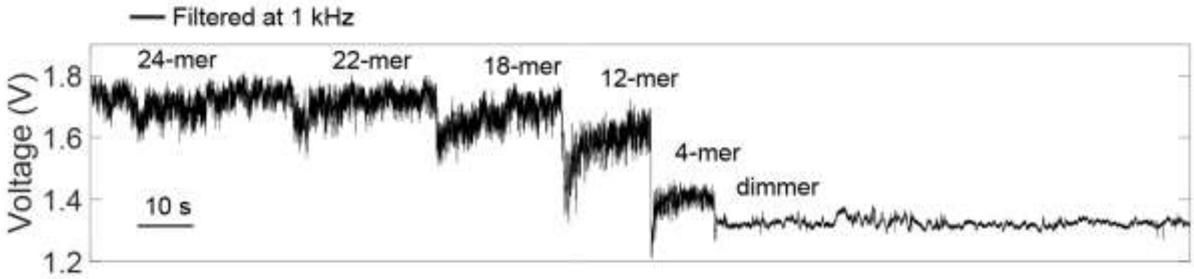

Figure S8. The whole trace of step-wise disassembly for test 2. Data was acquired at 1 MHz and filtered to 1 kHz. (DNH structure #3 used for this test– see Figure S1)

S9 – Stepwise disassembly trace of test 3

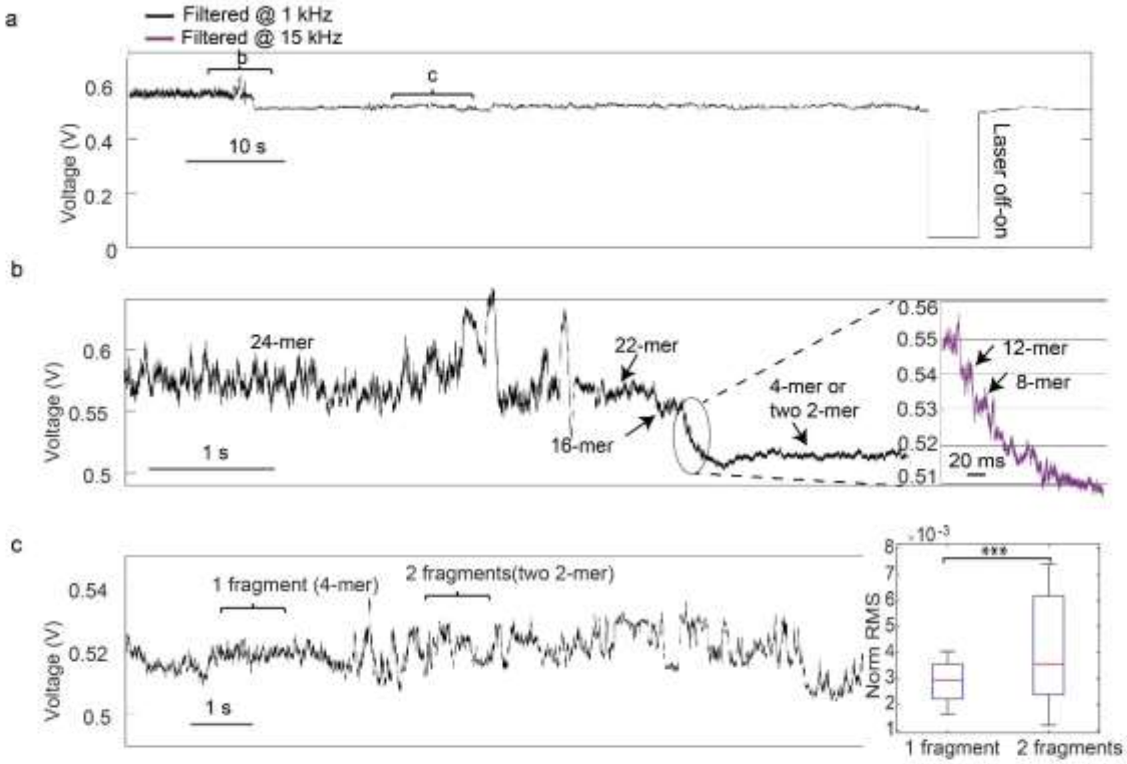

Figure S9. Disassembly trace of test 3. (a) Transmission trace showing the disassembly of ferritin at pH 2.0, encompassing turning off the laser to release the dimer fragments (two fragments) from the trap. (b) A zoomed-in view of the ferritin disassembly, highlighting the evident stepwise progression. The right panel image is an enlarged trace section, emphasising the disassembly steps. (c) Enlarged section of the transmission signal when two dimer fragments are both in the hotspot of the DNH. The right panel depicts the normalised RMS of the 2-second transmission segments marked in the trace, indicating the presence of one tetramer and two dimers in the trap. (DNH structure #4 used for this test– see Figure S1)

S10 - Stepwise disassembly trace of apo-ferritin

Figure S10 shows the impact of pH 2 on the disassembly process of apo-ferritin. To facilitate a more comprehensive comparison, single apo-ferritin molecule was trapped and exposed to acidic pH 2, revealing a stepwise disassembly pattern akin to that observed with holo-ferritin. This observed disassembly phenomenon is attributed to protonation occurring within the protein shell, thereby facilitating the disassembly process.[5]

Based on Figure S10b, the 24-mer ferritin dissociates into16-mer and 12-mer fragments. Following the 12-mer state, a sudden decrease in the transmission signal occurs (asterisk in Figure S10b). The observed decrease in transmission signal can be attributed to a shape change in the 12-mer ferritin fragments. This shape change likely affects the polarisability of the molecule, which in turn alters the transmission signal. However, the fragments appear to return to their initial conformation before ultimately disassembling into even smaller subunits.

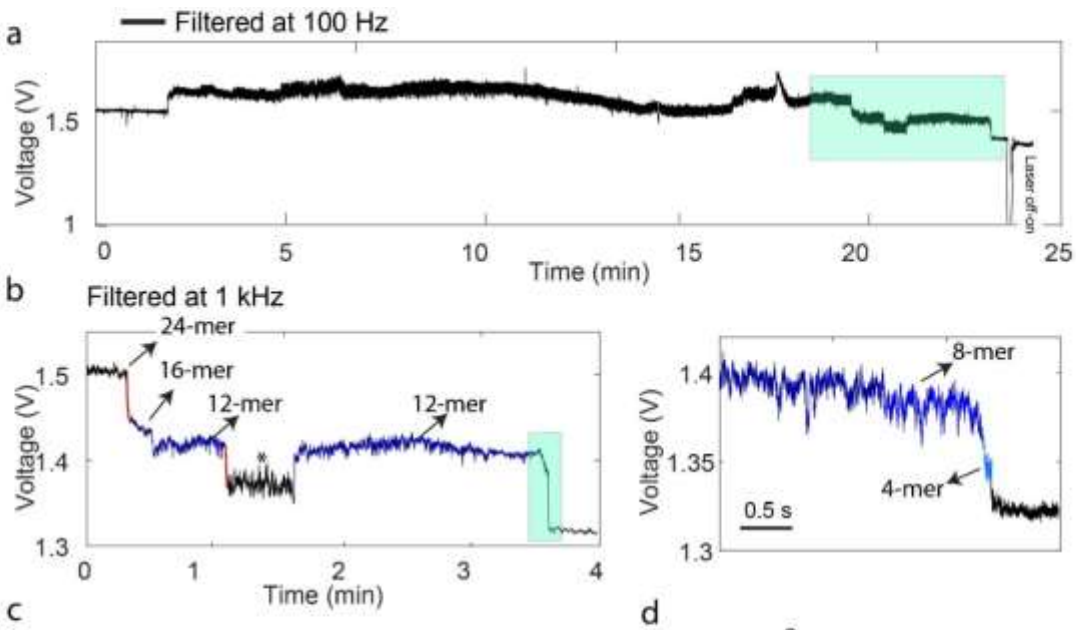

c

$V_{mean\,(mV)}$	$\frac{V_{xmer} - V_{DNH}}{V_{24mer} - V_{DNH}}$	*Ferritin Fragments*
1488	100%	24-mer
1425	65%	16-mer
1409	52%	12-mer
1381	37%	8-mer
1341	15%	4-mer
1317	0%	released

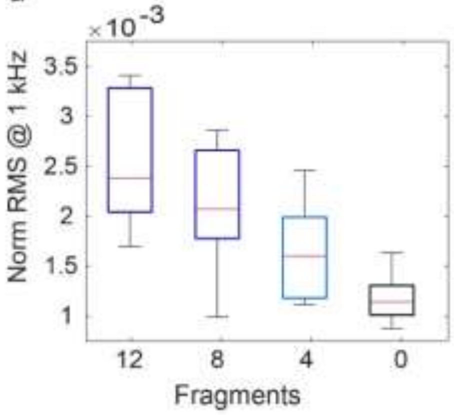

Figure S10. Single apo-ferritin molecule exposure to pH 2 (a) Continuous transmission trace of a DNH with an apo-ferritin protein trapped in pH 7.4 and subsequently subjected to pH 2. Following exposure to pH 2, the transmission signal gradually decreases over time, indicating a protein disassembly process. (b) The highlighted section in panel **a** displays the stepwise disassembly of 24-mer apo-ferritin into fragments. The right panel is also an enlarged disassembly step from the left panel (c) The table shows the mean values of steps and ferritin fragments that remained in the DNH trap. (d) Normalised RMS values related to the disassembly steps from 12-mer to when the DNH is empty. The data, Gaussian-filtered at 1 kHz, shows the 25th and 75th percentiles. (DNH structure #3 used for this test– see Figure S1). Raw data was collected at 1 MHz.

S11 - Size exclusion chromatography (protein markers)

To confirm the purity of ferritin at pH 7.4, the ferritin solution was examined by fast protein liquid chromatography (FPLC) with various protein markers. The molecular weight of these protein markers is outlined in the materials and method part and Figure S11. We also conducted this experiment with two variations: once including all protein markers and another time excluding ferritin. This was done to identify the peak position specifically associated with ferritin in neutral pH.

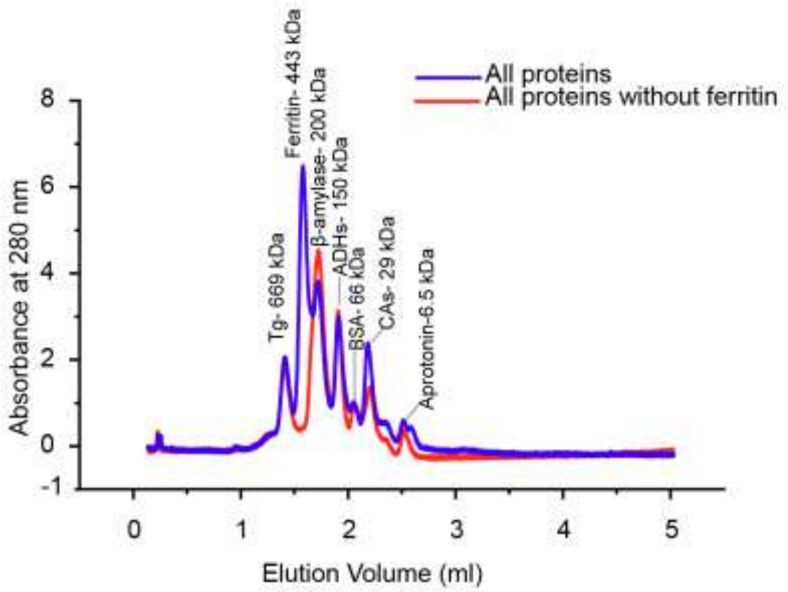

Figure S11. FPLC chromatogram of proteins markers including ferritin (blue), and marker proteins excluding ferritin (red) to confirm the peak position of ferritin.

S12 - Mass photometry

Single-molecule mass photometry (SMMP) is a label-free technique that assesses the molecular mass of individual molecules, particularly biomolecules ranging from 40 kDa to 5 MDa within a sample. The alteration in reflectivity at the glass-water interface, induced by the binding of a biomolecule from the solution to the coverslip surface, leads to a localised change in contrast.[6] SMMP results in Figure S12 display peaks representing the native 24-mer assembled ferritin in T50 buffer at pH 7.4. Additionally, a broad peak of the 48-mer is evident in the figure, suggesting the potential binding of two ferritin molecules together. Furthermore, distinct fragments around 60 kDa, separate from the fully assembled ferritin, are evident in the figure.

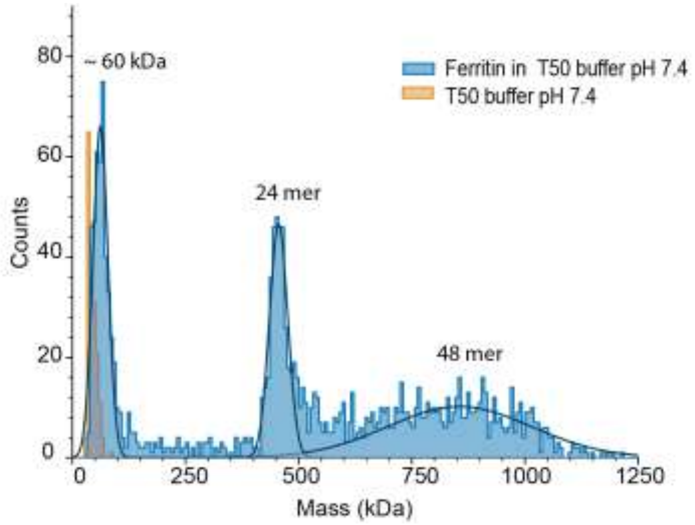

Figure S12. MP of T50 buffer pH 7.4 with and without 50 nM ferritin.

References

(1) Yousefi, A.; Ying, C.; Parmenter, C. D. J.; Assadipapari, M.; Sanderson, G.; Zheng, Z.; Xu, L.; Zargarbashi, S.; Hickman, G. J.; Cousins, R. B.; Mellor, C. J.; Mayer, M.; Rahmani, M. Optical Monitoring of In Situ Iron Loading into Single, Native Ferritin Proteins. *Nano Lett* **2023**, *23* (8), 3251–3258. https://doi.org/10.1021/acs.nanolett.3c00042.

(2) Ghorbanzadeh, M.; Jones, S.; Moravvej-Farshi, M. K.; Gordon, R. Improvement of Sensing and Trapping Efficiency of Double Nanohole Apertures via Enhancing the Wedge Plasmon Polariton Modes with Tapered Cusps. *ACS Photonics* **2017**, *4* (5), 1108–1113. https://doi.org/10.1021/acsphotonics.6b00923.

(3) Badu-Boateng, C.; Pardalaki, S.; Wolf, C.; Lajnef, S.; Peyrot, F.; Naftalin, R. J. Labile Iron Potentiates Ascorbate-Dependent Reduction and Mobilization of Ferritin Iron. *Free Radic Biol Med* **2017**, *108*, 94–109. https://doi.org/10.1016/j.freeradbiomed.2017.03.015.

(4) Takeda, S.; Yamaki, M.; Ebina, S.; Nagayama, K. Site-Specific Reactivities of Cysteine Residues in Horse L-Apoferritin. *J Biochem* **1995**, *117* (2). 267-270. https://doi.org/10.1093/jb/117.2.267.

(5) Kim, M.; Rho, Y.; Jin, K. S.; Ahn, B.; Jung, S.; Kim, H.; Ree, M. PH-Dependent Structures of Ferritin and Apoferritin in Solution: Disassembly and Reassembly. *Biomacromolecules* **2011**, *12* (5). 1629-1640. https://doi.org/10.1021/bm200026v.

(6) Foley, E. D. B.; Kushwah, M. S.; Young, G.; Kukura, P. Mass Photometry Enables Label-Free Tracking and Mass Measurement of Single Proteins on Lipid Bilayers. *Nat Methods* **2021**, *18* (10). 1247-1252. https://doi.org/10.1038/s41592-021-01261-w.
